# Chlorogenic acid effectively treats cancers through induction of cancer cell differentiation

**DOI:** 10.7150/thno.34674

**Published:** 2019-09-19

**Authors:** Shuai Huang, Lu-Lu Wang, Ni-Na Xue, Cong Li, Hui-Hui Guo, Tian-Kun Ren, Yun Zhan, Wen-Bing Li, Jie Zhang, Xiao-Guang Chen, Yan-Xing Han, Jin-Lan Zhang, Jian-Dong Jiang

**Affiliations:** 1 State Key Laboratory of Bioactive Substance and Function of Natural Medicines, Institute of Materia Medica, Chinese Academy of Medical Sciences and Peking Union Medical College, Beijing, 100050, China; 2 Department of Glioma, Beijing Shijitan Hospital, Capital Medical University, Beijing, 100038, China; 3 Jiuzhang Biochemical Engineering Science and Technology Development Co., Ltd, Chengdu, Sichuan, 610041, China; 4 Laboratory of Antiviral Research, Institute of Medicinal Biotechnology, Chinese Academy of Medical Sciences and Peking Union Medical College, Beijing, 100050, China

**Keywords:** chlorogenic acid, cancer differentiation, SUMO1, c-Myc, p21

## Abstract

Rationale: Inducing cancer differentiation is a promising approach to treat cancer. Here, we identified chlorogenic acid (CA), a potential differentiation inducer, for cancer therapy, and elucidated the molecular mechanisms underlying its differentiation-inducing effects on cancer cells.

Methods: Cancer cell differentiation was investigated by measuring malignant behavior, including growth rate, invasion/migration, morphological change, maturation, and ATP production. Gene expression was analyzed by microarray analysis, qRT-PCR, and protein measurement, and molecular biology techniques were employed for mechanistic studies. LC/MS analysis was the method of choice for chemical detection. Finally, the anticancer effect of CA was evaluated both *in vitro* and *in vivo.*

Results: Cancer cells treated with CA showed reduced proliferation rate, migration/invasion ability, and mitochondrial ATP production. Treating cancer cells with CA resulted in elevated SUMO1 expression through acting on its 3'UTR and stabilizing the mRNA. The increased SUMO1 caused c-Myc sumoylation, miR-17 family downregulation, and p21 upregulation leading to G_0_/G_1_ arrest and maturation phenotype. CA altered the expression of differentiation-related genes in cancer cells but not in normal cells. It inhibited hepatoma and lung cancer growth in tumor-bearing mice and prevented new tumor development in naïve mice. In glioma cells, CA increased expression of specific differentiation biomarkers Tuj1 and GFAP inducing differentiation and reducing sphere formation. The therapeutic efficacy of CA in glioma cells was comparable to that of temozolomide. CA was detectable both in the blood and brain when administered intraperitoneally in animals. Most importantly, CA was safe even at very high doses.

Conclusion: CA might be a safe and effective differentiation-inducer for cancer therapy. “Educating” cancer cells to differentiate, rather than killing them, could be a novel therapeutic strategy for cancer.

## Introduction

Cell differentiation is a programmed maturation of the immature stage which alters cell structure and function. The switch toward differentiation requires coordinated regulation of the expression of a group of genes at the post-transcriptional level involving epigenetic and/or miRNA-mediated mechanisms 1, 2. As the main characteristic features of cancer cells are sustained proliferation and vigorous migration, induction of cancer cell differentiation and correction of malignant behavior might constitute one of the ideal approaches for cancer treatment. Differentiated cancer cells have low proliferation rate, reduced mobility, restricted ATP production and normalized cell morphology as compared to the undifferentiated ones 3-5. However, studies of differentiation inducers, such as those of dimethyl sulfoxide (DMSO), arabinosylcytosine and actinomycin D, are stymied partially by their side effects 6. Successful drugs in this field include arsenic trioxide (As_2_O_3_, ATO) and all-trans retinoic acid (ATRA). ATO induced cell differentiation in acute promyelocytic leukemia (APL) by, at least in part, attacking the PML moiety of the PML-RARα fusion products 7, whereas ATRA-stimulated APL cell differentiation through interacting with the nuclear receptor RAR 8, 9. Both drugs showed remarkable results in treating APL patients when used alone. In combination, the two could cure APL in many patients shedding new light on anticancer therapy 10-12. Clinically, ATO and ATRA are effective mainly for specific types of leukemia and their side effects are related to heart, liver, and kidney 13. Discovery of safe differentiating agents for solid cancers is highly desirable for the successful implementation of this approach in cancer treatment.

Although plants replace their foliage each year, placing an immensely heavy load on plant cell biosynthesis, it has been shown to be non-tumorigenic and plants very rarely develop cancer 14. The rigid physical constraint by plant cell wall might be a contributing factor 14; however, plants might possess innate mechanisms to control the meristematic tissue, such that they maintain the standard cycle of division-to-differentiation without erroneously progressing to malignancy. A challenging task in developmental biology is to learn the detailed regulatory mechanism of meristem maintenance and the transition to a differentiated cell. The process could include recruitment of cells within the meristem, cell division into primordium, post-primordial division, and differentiation into the mature plant organs, such as the leaf, flower, etc. 15. Induction of differentiation in plants is associated with the plant's regulatory peptides and metabolites, which bring inter-meristematic communication and cell to cell interaction 16, 17. For example, the phytohormone auxin influx causes the root protophloem differentiation response 18, 19; plants also synthesize plenty of ascorbate as a cofactor with Fe^2+^/α-KG-dependent dioxygenases in the chloroplast of the meristem to control cell differentiation through epigenetic mechanisms 20. Besides, reactive oxygen species (ROS) signaling cascades might be involved in plant cell differentiation 21. Considering that plants have no immune system 22 with which cancer could be eliminated, an effective and well-established differentiation mechanism must be in place to correct malignant growth.

**Figure 1:**
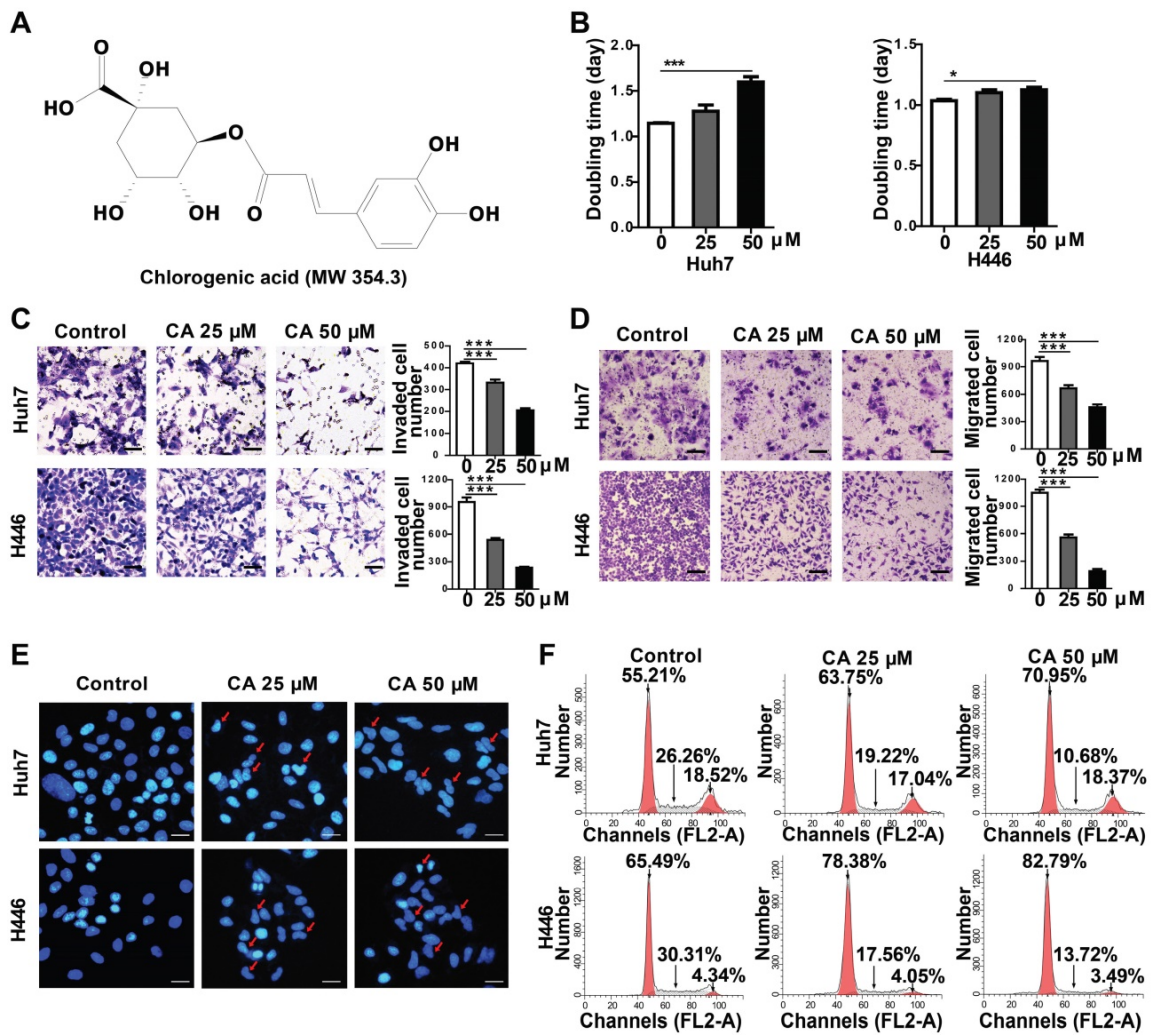
** Effect of CA on Huh7 and H446 cells. (A)** Chemical structure of CA. **(B)** Increased doubling time of Huh7 and H446 cells after treatment with CA (25 or 50 µM) on day-4. **(C)** The representative image of invading cells after exposure to CA (25 or 50 µM; 24 h. Scale bars indicate 10 μm). **(D)** The representative image of migrating cells after exposure to CA (25 or 50 µM; 24 h. Scale bars indicate 10 μm). **(E)** CA-induced morphological change in cellular nuclei (CA at 25 or 50 µM; 48 h). Red arrow indicates cell karyopyknosis (stained with DAPI; 400 ×; Bar, 10 μm). **(F)** Cell cycle analysis of Huh7 and H446 cells treated with CA (25 or 50 μM for 24 h). Compared with the solvent control, the number of cells in the G0/G1 phase increased from 55.21% to 63.75% (CA 25 μM) or 70.95% (CA 50 μM) in Huh7 cells, and from 65.49% to 78.38% (CA 25 μM) or 82.79% (CA 50 μM) in H446 cells. S-phase cells decreased from 26.26% to 19.22% (CA 25 μM) or 10.68% (CA 50 μM) in Huh7 cells and from 30.31% to 17.56% (CA 25 μM) or 13.72% (CA 50 μM) in H446 cells. All data are expressed as mean ± SEM. The experiments were done 3 times. * p < 0.05, *** p < 0.001.

Several botanic compounds have been found to actively induce differentiation in mammalian cells, such as retinoic acid, matrine, shikonin, and cotylenin A, among others 8, 23-25. Chlorogenic acid (CA) is a botanic compound (mw 354.31, Figure 1A) isolated from plants such as *Eucommia almoides oliver* or *Lonicera confuse* 26. Chemically, it belongs to the phenylpropanoids with polyhydroxyphenol groups and has been reported to be non-toxic in animals and safe in humans 27. The compound seems to be active against oxidation and inflammation 28, 29, and has some regulatory activity on the immune system 30. Recently, CA has been identified to be a potential drug for cancer and was approved by the China Food and Drug Administration (CFDA) first for phase I (NCT02728349, Apr. 2016) and then phase II (NCT03758014, Nov. 2018) clinical trials in glioma patients. Although the anticancer molecular mechanism of CA is far from clear, its unique effects against cancer provoked our interest to include CA in our investigation of potential botanic differentiation inducers. We found that CA treatment altered tumor cell behavior and programmed cancer cell differentiation both* in vitro* and *in vivo*. Human solid tumor cell lines from hepatoma, lung cancer, glioma, and colon cancer exhibited a similar response to CA. Here, we present data suggesting that CA is a novel and safe differentiation inducer for the treatment of solid tumors.

## Methods

### Chemicals

Chlorogenic acid (CA, Figure 1A) with a purity of more than 99% was acquired by the Jiuzhang Biochemical Engineering Science and Technology Development Co., Ltd. (Chengdu, Sichuan, China). For the *in vitro* experiments, CA was dissolved in dimethyl sulfoxide (DMSO, Sigma Aldrich, St. Louis, USA) at a concentration of 100 mM as a stock solution, followed by dilution in medium (or saline) prior to the experiments. DMSO was used as a vehicle reference in the study (as an untreated control group).

### Cell lines, culture, and transfection

Human NCI-H446, Huh7, Bel-7402, HEK293T, HCT-116, U87MG, and M059J cells were from the American Type Culture Collection (ATCC, MD, USA); the CCC-HEL-1, NCI-H358, WI-38, MRC-5, A549-5FU, SK-LU-1, rat C6, and mouse G422 glioma cells were from the Cell Center, Peking Union Medical College (Beijing, China); the MIHA cells were kindly provided by Dr. Chen YC of the Chinese University of Hong Kong (Hong Kong, China); the iPS cells were from the Cellapybio Company (Beijing, China); the HH were purchased from the ScienCell Research Laboratories (San Diego, CA, USA).

The Huh7, M059J, CCC-HEL-1 and HEK293T cells were cultured in the Dulbecco's Modified Eagle's Medium (Invitrogen, CA, USA) supplemented with 10% fetal calf serum (FBS, Invitrogen), penicillin (100 U/mL) and streptomycin (100 μg/mL) (Invitrogen). The NCI-H446, NCI-H358, Bel-7402 and MIHA cells were cultured in the RPMI-1640 medium (Invitrogen) supplemented with 10% FBS, penicillin and streptomycin (P/S, Invitrogen). The SK-LU-1, U87MG, SF-126, WI-38, and MRC-5 cells were cultured in Minimum Essential Medium (Invitrogen) supplemented with 10% FBS and P/S. The HCT-116 and A549-5FU cells were cultured in McCoy 5'A medium (Invitrogen) supplemented with 10% FBS and P/S. The human iPS cells were cultured in PSCeasy medium (Cellapybio), which was changed every day for the maintenance of stemness. Human hepatocytes (HH) were cultured in hepatocyte medium (ScienCell) with 5% FBS (ScienCell) and 1% hepatocyte growth supplement (ScienCell). All cell lines were cultured in a humidified atmosphere containing 5% CO_2_ and maintained at 37 °C.

For the regulation of expression, cells were seeded the day before transfection into 12-well plates with antibiotic-free growth medium at 1 × 10^6^ cells/well and cultured overnight until they reached 60-70% confluence. Transfection of the negative control (50 nM), mimics (50 nM), inhibitors (100 nM) and/or siRNAs (50 nM) was carried out using riboFECT™ CP transfection reagent (Ribobio, Guangzhou, China) for 24 h according to the manufacturer's protocol. The siRNAs used in this study were synthesized by Ribobio (Guangzhou, China).

### Cell staining

Cells were rinsed three times in PBS and fixed with cold methanol, washed thoroughly with PBS, incubated with DAPI staining solution (1 µg/mL, Thermo Fisher Scientific, Waltham, MA, USA) for 30 minutes (min) and rinsed three times with PBS, followed by viewing using a fluorescence microscope (Olympus, Tokyo, Japan).

For the immunofluorescent staining, the human glioma cells U87MG and M059J were cultured on the coverslips and treated with CA (25 µM, 50 µM) for 24 h, Subsequently, the cells on the coverslips were fixed with 4% paraformaldehyde (15 min at 4 °C), blocked with 1% BSA (1 h, RT), and incubated with primary antibody, rabbit anti-GFAP antibody (1:200 dilution; Proteintech, IL, USA), or mouse anti TUBB3-antibody (Tuj1, 1:200 dilution; Proteintech, IL, USA) for overnight at 4 °C. The coverslips were then washed 3 times with PBS and stained with a Cy3-conjugated anti-mouse secondary antibody or an Alexa Fluor 488-conjugated anti-rabbit secondary antibody (1:200 dilution; Thermo Fisher Scientific, Waltham, MA, USA). Cell nuclei were counterstained with DAPI (1 µg/mL, Thermo Fisher Scientific, Waltham, MA, USA). Confocal images were taken using a fluorescent microscope (Nikon Eclipse CI, Japan) and fluorescence pictures were photographed using the Nikon DS-U3 system (Japan).

### Cell viability and growth assay

Cell growth was examined using the 3-(4,5-dimethylthiazol-2-yl)-2,5-diphenyltetrazolium bromide (MTT, Thermo Fisher Scientific, Waltham, MA, USA) assay for which 10^4^ cells were seeded into each well of a 96-well plate. For growth analysis, the cells were treated with blank or 25 or 50 µM of CA every 24 h for 6 days. At different time points, the cells were stained with 50 μL of MTT reagent at 37 °C for 4 h. Spectrometric absorbance at 550 nm was detected using a microplate reader (BioTek, Vermont, USA). The doubling-time (Td) of the tumor cells was calculated using the following formula:

Td = t*[lg2/ (lgNt-lgNo)], where Nt is the cell number after treatment for 4 days.

### Migration and invasion assay

Cells were treated with CA (25 μΜ or 50 μΜ) for 24 h, trypsinized and resuspended in serum-free medium. Next, 100 μL of the cell suspension (1 × 10^5^ cells) was seeded into the upper chamber of the Transwell (Corning Costar, MA, USA) with a porous polycarbonate membrane (pore diameter of 8 μm). The lower chamber contained 700 μL of medium supplemented with 10% FBS. After 24 h incubation, the cells on the upper surface of the membrane filters were mechanically removed with cotton swabs. Migrated cells on the lower side of the filter were fixed with cold methanol, stained with 0.5% crystal violet solution for 120 min, washed with PBS, counted, and captured under a microscope. For the cell invasion assay, the upper chamber of the Transwell was coated with 60 µL of Matrigel (1:20 Corning Costar, MA, USA), and cells that invaded the lower surface of the membrane filter were analyzed as described above.

### Vector construct

Oligonucleotide fragments, including the target region or mutant target region of the miRNA seed sequence AAGUGC, were synthesized (Invitrogen, CA, USA), annealed and ligated into the pmiRGlO dual luciferase miRNA target expression vector (Promega, WI, USA), and labeled pmiRGlo p21 position 1 (P1), position 2 (P2), pmiRGlo p21 mutant position 1 (mP1), or mutant position 2 (mP2). The EcoRV internal restriction site was used for clone confirmation. The bands were separated on a 1% agarose gel, stained with SYBR Green Safe and visualized under the UV trans-illuminator (Bio-Rad, Hercules, CA, USA).

### Dual luciferase reporter assay

HEK293T cells, commonly used for the dual luciferase reporter assay were transfected with miR-20a, -93, -106b mimics (50 nM), or vector constructs (200 ng) using FuGENE^@^ HD transfection reagent (Promega) for 24 h. The cells were then added to 100 μL of Passive Lysis Buffer (PLB) and gently mixed by shaking for 15 min. The lysate was immediately measured using the Dual-Luciferase^@^ Report Assay System (Promega). Data were presented as a ratio of Firefly to Renilla luciferase activity (Fluc/Rluc). All assays were performed three times in triplicate.

### Quantitative real-time PCR (qRT-PCR)

Total RNA was extracted from cells or tissues using the SV Total RNA Isolation System (Promega Biotech, Madison, USA). Subsequently, 2 μg of RNA was used for reverse transcription and the quantitative PCR reaction according to the manufacturer's instructions (Invitrogen). Mature miR-20a, -93 and -106b levels were measured using the Bulge-Loop miRNA qRT-PCR kit (Ribobio, Guangzhou, China) with U6 snRNA serving as an internal control. The resulting cDNA products were analyzed with the quantitative real-time PCR 7500 fast system (Applied Biosystems, Foster, USA). Total RNA (100 ng) and one-step power SYBR reagent (Invitrogen) were used for the detection of mRNAs. The mRNA expression of the housekeeping gene GAPDH was used for normalization. The primer sequences used in this study are listed in Table S1. Data analysis was performed using the 2^-ΔΔCt^ method.

### Western blotting

Total proteins were extracted from cells or tissues using the M-PER mammalian protein extraction reagent (Thermo Pierce, Rockford, USA) and measured using the BCA Protein Assay (Thermo Pierce). Protein samples (50 μg per assay) were separated on SDS-PAGE gels and transferred to PVDF membranes. The membranes were blocked with 5% skim milk and then incubated overnight at 4 °C in the presence of primary antibodies. The primary antibodies for p21, p53, KHSRP, c-Myc, EPCAM, and *β*-actin were purchased from Cell Signaling Technologies (CST, Danvers, MA, USA), *p*-c-Myc antibody was from Santa Cruz (CA, USA), and I*κ*B*α*, Rb1, STAT3, histone-H3, SUMO1, and SUMO2/3 antibodies were from the Proteintech Company (Chicago, IL, USA). The membranes were washed 3 times in Tris-buffered saline with Tween-20 and incubated with horseradish peroxidase-conjugated secondary antibody (CST, MA, USA) for 1 h at room temperature (RT). After washing 3 times with 15 mL of TBST, Immobilon Western Chemiluminescent HRP Substrate (Millipore, Billerica, USA) was added, and the bands were imaged with the Chemidoc XRS^+^ electrophoretic imaging system (Bio-Rad, Hercules, CA, USA). Density scanning of each protein band was performed using Image Lab software (Bio-Rad, Hercules, CA, USA).

### Flow cytometry analysis

Cells were harvested 24 h after treatment with DMSO (solvent control) or CA (25 μM or 50 μM) and rinsed twice with PBS. The cells were fixed with cold ethanol, washed with PBS twice, and centrifuged at 300 × *g* for 5 min. Next, 500 μL of propidium iodide (50 μg/mL) was added to each sample tube, followed by incubation for 30 min at 37 °C in the dark. The cell cycle distribution was analyzed using a C6 Flow Cytometer (BD FACSCalibur, Ann Arbor, MI). For CD44 expression, 1 μL of PE-conjugated CD44 antibody (1:100, Miltenyi Biotec, Cologne, Germany) was added to the cells, followed by a 30 min incubation, washing, and re-suspension in 500 μL of PBS for analysis.

### Sumoylation assay

Nuclear proteins were extracted from sample cells or tissues using NE-PER nuclear and cytoplasmic protein extraction reagent (Thermo Pierce, USA) and measured with the BCA Protein Assay (Thermo Pierce). The sumoylation assay was performed using the EpiQuikTM Protein Sumoylation Assay Ultra kit (Epigentek, #P-8003, Farmingdale, USA). The standard procedure was as follows: primary antibody (IP-grade) or control was added to coat the microplate, which was evaporated at 37 °C for 120 min and the unoccupied sites were blocked with blocking buffer for 45 min at RT. SUMO assay buffer (SAB) was added to each well. Next, sample nuclear proteins (10 μg) were added to each sample well, the diluted detection antibody against SUMO proteins was added to each well for 60 min at RT and then color developer was added and incubated for 5 min. After the addition of stop solution, the absorbance was measured using a microplate reader (BioTek, USA).

### Transcriptome and miRNA microarray expression profiling

Total RNA was extracted from CA-treated Huh7 cells, and the samples were used for whole human genome oligo microarray and miRNA microarray profiling. For the transcriptome microarray, RNA sample labeling and microarray hybridization were performed according to the Agilent One-Color Microarray-Based Gene Expression Analysis protocol (Agilent Technology). Briefly, total RNA was linearly amplified, labeled with Cy3-UTP, and then hybridized onto the microarray chips (4 × 44 K, Agilent, Santa Clara, CA, USA). After washing and staining the slides, the arrays were scanned using the Agilent Microarray Scanner (part number G2505C). The scanned images were analyzed using Agilent Feature Extraction Software (version 11.0.1.1).

The miRNA expression was measured by the Agilent microarray according to the standard Agilent microarray scanning process. The miRNA labeling was performed using the miRCURY™ Hy3™/Hy5™ Power labeling kit (Exiqon, Vedbaek, Denmark). The Hy3™-labeled samples were hybridized onto the miRCURYTM LNA Array (Exiqon, v.18.0), and the slides were scanned using the Axon GenePix 4000B microarray scanner (Axon Instruments, Foster City, CA). The scanned images were analyzed using GenePix Pro 6.0 software (Axon).

### SUMO1 mRNA stabilization

The *SUMO1* mRNA 3' UTR region has a total length of 1073 bp and contains 4 AU-rich elements (AREs). We acquired two fragment sequences containing ARE1-3 (nt 455-1222) and ARE4 (nt 1443-1457) using Nhel-tailed forward and Xbal-tailed reversed primers. The three consecutive fragments were individually inserted into the pIS0 vector and pRL-TK containing Renilla luciferase was used as an internal plasmid. The Huh7 and H446 cells were transiently transfected with the pIS0-SUMO1-3' UTR (nt 455-1527), pIS0-SUMO1-3' UTR-Del-ARE4 (nt 455-1284), and pIS0-SUMO1-3' UTR-Del-ARE1-3 (nt 1285-1527). After 4 h of transfection, the cells were treated with or without 50 μΜ of CA for 20 hr, followed by the dual luciferase reporter assay. Data were represented as a ratio of firefly to Renilla luciferase activity (Fluc/Rluc).

### Sphere formation assay

Neurosphere test was performed in the U87MG and M059J glioma cells using the previously described method 31. 10^3^ cells were seeded into each well of the low-attachment 6-well plates (Corning Costar, MA, USA). A final concentration for 20 ng/mL EGF (Gibco, USA), 10 ng/mL bFGF (Gibco, USA), 10 μL/mL B27 (Gibco, USA), and 5 μg/mL Insulin (Gibco, USA) was added to the culture medium DMEM/F12 (Gibco, USA). Sphere formation was examined after 14 days of CA treatment.

### ATP and OCR measurement

Cellular ATP content was determined using the EnzyLight^TM^ ATP assay kit (EATP100, Hayward, CA, USA) and operated according to the manufacturer's instructions. Briefly, 10^4^ cells (per well) were seeded into the white opaque 96-well plate (Corning Costar, MA, USA) followed by transfection with negative control or *SUMO1* siRNA in the presence or absence of CA (50 μM). Following 24 h incubation, the luminescence was measured after adding the reconstituted reagents of the kit, and the ATP concentration was calculated based on a calibration plot made with standard ATP solution (ranging from 3 μM to 30 μM).

Cellular oxygen consumption rate (OCR) test was performed using an XF24 Seahorse Analyzer (Seahorse Bioscience, North Billerica, MA, USA). Briefly, 2×10^4^ cells were seeded into each well of the XF24 cell culture microplate with freshly prepared growth medium for 16 h and then treated with CA (0, 25, 50 μM) for another 24 h. The OCR value was detected with the XF Cell Mito Stress Test Kit (Seahorse Bioscience, North Billerica, MA, USA) according to the operation manual instructions. The mitochondrial inhibitors used were 1 μM oligomycin A, 1 μM carbonyl cyanide p-trifluoro-methoxyphenyl hydrazine (FCCP), and 0.5 μM Rotenone plus 0.5 μM antimiycin A (Ron/AA). All OCR values were normalized to the protein content (μg/μL) of each well, which were detected with the BCA protein assay kit.

### Xenograft tumor model

Male NOD/SCID mice, weighing 18-22 g, were purchased from Vital River Laboratories (Beijing, China) and housed with free access to food and water. All experimental animal procedures were approved by the Animal Experimental Ethics Committee of the Institute of Materia Medica, Chinese Academy of Medical Sciences and Peking Union Medical College. To establish the mouse xenograft model, Huh7 or H446 cells (2 × 10^7^ cells/mouse) in Matrigel-containing culture medium were subcutaneously inoculated into the mice. The tumor-bearing mice were treated with CA when the xenograft tumor mass reached 100 mm^3^. The animals (7 per group) received an intraperitoneal (ip) injection of CA (directly dissolved in saline; 25, or 50, or 200 mg/kg/d) for 30 days, and the same volume of saline was used as a control. To terminate the experiment, the body weights of the mice were determined. The tumor volume (TV) was calculated with electronic calipers using the following formula: TV = (a × b^2^)/2 (where “a” is the largest superficial diameter and “b” is the smallest superficial diameter). The tumor inhibition rate (TIR, %) was calculated using the following formula: TIR = (the mean tumor weight (volume) of the control group - the mean tumor weight (volume) of the treated group) / the mean tumor weight (volume) of the control group × 100%. After sacrificing the mice, the tumor weights were determined, and the tumor tissue samples were collected for qRT-PCR and Western blotting. The tumor re-implantation test was carried out in which 25 mg of tumor tissues from each group was pooled, enzyme digested, homogenized, and diluted at 1:4 and 1:8 ratios in culture medium followed by reimplantation of the tumor cell suspension into naïve NOD/SCID mice (2 × 10^5^ or 1 × 10^5^ cells per mouse for the 1:4 or 1:8 dilutions, respectively). Tumor growth in the mice was monitored for 4 weeks.

### Safety assessment of CA in vivo

KM mice were purchased from the Vital River Laboratories (Beijing, China) and randomly divided into 4 groups. Animals (n = 10 per group, ♀ × 5, ♂ × 5) were treated intraperitoneally with a single dose of 1000, or 500, or 250 mg/kg CA, and saline was used as a control. Mouse survival and body weight changes were examined daily for all groups for 7 days. Next, 200 µL blood samples were collected from each mouse via retro-orbital bleeding, and levels of ALT, AST, BUN, and CRE in serum were detected.

### CA concentration in animal blood and brain

Wistar rats (male, 250 ± 20 g) were acquired from Vital River Laboratories (Beijing, China). CA in saline was administered to the rats (ip, 75 mg/kg) and three rats per time point were exsanguinated at 1, 2, 4, 7, 10, 20, 30, 45, 60, and 120 min post-injection. Blood was collected into a heparinized test tube and centrifuged at 4500 rpm for 10 min. Plasma was stored at -80 °C and thawed at room temperature prior to use. For analysis, 20 μL of CA (2.5-5000 ng/mL an equivalent gradient concentrations), 20 μL of puerarin (25 ng/mL, internal standard, Baoji Herbest Bio-Tech, Shanxi, China) and 20 μL of 2 M HCl was added to 100 μL of plasma followed by 700 μL of acetonitrile. The mixture was vortex-mixed for 5 min, centrifuged at 4500 rpm for 10 min, and the supernatant acetonitrile extraction solution was evaporated to dryness with a gentle stream of nitrogen gas at 30 °C. The residue was reconstituted in 50 μL of the initial mobile phase by vortexing for 3 min and centrifuged at 13000 rpm for 10 min. Finally, 5 μL of supernatant was injected into the UPLC-MS/MS system (Agilent Corporation, MA, USA). Values were presented as the mean ± SD of 3 rats per time point. To determine the pharmacokinetic parameters of chlorogenic acid, the concentration-time data were analyzed using the DAS (Drug and Statistics for Windows) software package (version 2.0, Chinese Pharmacological Association, China).

To measure CA in rat brain, CA in saline was ip administered to the Wistar rats, with 3 rats for each group. At 1, 2, 4, 7, 10, 20, 30, 45, 60, 120, 180, 300, and 420 min post-CA injection, the rats were administered with pentobarbital sodium (ip, 60 mg/kg) to induce deep anesthesia, followed by surgery and injection of saline into the ventricle to remove blood from the blood vessels. Rat brain was then homogenized, and the CA concentration in the brain was determined using a similar chemical detection method in the UPLC-MS/MS system as mentioned above.

### Treating glioma with CA in animals

For the C6 glioma in rats, CA (purity > 99%) was from the Jiuzhang Biochemical Engineering Science and Technology Development Co., Ltd. (Chengdu, Sichuan, China). Temozolomide (TMZ, purity over 98%) was purchased from Sigma-Aldrich (St. Louis MO, USA). The rat glioma C6 cells were obtained from the Cell Center, Peking Union Medical College, and cultured in DMEM with 10% FBS, penicillin (100 units/mL) and streptomycin (100 μg/mL). Male Wistar rats (260-280 g) were from Vital River Laboratories (Beijing, China).

Before the surgical procedures, male Wistar rats were anesthetized by an intraperitoneal injection of pentobarbital sodium (60 mg/kg). The rat head was shaved and disinfected with 70% ethyl alcohol, and then the animal was fixed in a stereotactic apparatus. A midline incision was generated on the dorsal aspect of the rat head to expose the bregma. A burr hole was then generated in the cranial bone 0.2 mm anterior to the bregma and 3 mm lateral to the sagittal suture. A 25 μL Hamilton microsyringe was connected to the manipulating arm of the stereotactic device. The tip of the microsyringe was inserted 7 mm beneath the dura and then withdrawn 1 mm. Next, 10 μL of C6 glioma cells (4 × 10^5^) were injected stereotactically into the striatum. The injection was conducted over 10 min, after which the syringe was maintained in the brain for another 10 min and then slowly retracted. The wounds were cleaned, and the animals were then returned to their cages and received the standard rat diet and water ad libitum.

After 11 days of inoculation, 18 rats developed tumors based on magnetic resonance imaging (MRI) scanning using the Bruker PharmScan system (Bruker Biospin, Karlsruhe, Germany). The tumor-bearing rats were then randomly assigned to three treatment groups (n = 6), including the model group, TMZ group and CA group. Another six rats were designated as the sham-operated group (Sham group), which received an injection of 10 μL of cell culture medium without glioma cells using the same surgical procedure. The rats in the TMZ group were orally treated with a solution of TMZ (20 mg/kg/d) for five consecutive days, and those in the CA group were intraperitoneally administered CA (75 mg/kg, bid) for seven consecutive days. The body weights of the rat were measured every day. The tumors were scanned by MRI on days 11 and 18 after tumor cell injection. The experiment was then terminated after the second MRI scan.

### Statistical analysis

Heterogeneity of variance among groups was first examined by F-tests, followed by difference analyses performed with the unpaired 2-tail Student's *t*-test using GraphPad Prism 5.0 software (San Diego, CA, USA), p < 0.05 was considered significant.

## Results

### CA changed tumor cell behavior by inducing differentiation *in vitro*

The effect of CA was investigated in a variety of solid tumor lines, such as human hepatoma (Huh7) and lung cancer (H446) cells, for its anticancer effects. Compared with the untreated groups, CA slowed down the proliferation rate of tumor cells. In the Huh7 cells, the doubling time (Td) on day 4 of treatment was extended from 1.15 to 1.28 days at 25 µM CA concentration and up to 1.60 days at 50 µM. For the H446 cells, Td was extended from 1.04 to 1.10 (CA 25 µM) and 1.12 days (CA 50 µM) (Figure 1B). The cell mobility assay showed that the number of invading (Figure 1C) and migrating cells (Figure 1D) was significantly lower in the Huh7 and H446 cells treated with CA (25 or 50 µM for 24 h) as compared with the untreated controls. CA appeared to reduce the tumor cell growth rate and inhibited cell mobility through a non-cytotoxic mechanism. Figure 1E shows the morphological changes in the Huh7 and H446 cells after exposure to CA. With respect to the untreated cells, the cell nuclei underwent karyopyknosis and exhibited a kidney shape after treatment with 25 or 50 μM of CA for 48 h. This phenotypic change suggested a transition toward cell maturation. Cancer cell differentiation was also supported by the cell cycle arrest at the G_0_/G_1_ phase after treating the Huh7 or H446 cells with CA (25 or 50 μM for 24 h). Compared with the solvent controls, the percent of cells in the G_0_/G_1_ phase increased, and S-phase cells decreased in the two cell lines indicating a slowed-down biosynthesis (Figure 1F) and cell maturation 32. The G_2_/M phase remained stable. Human glioma cells showed a similar response to CA (see the glioma section below).

### CA regulated expression of the differentiation-related genes

**Figure 2:**
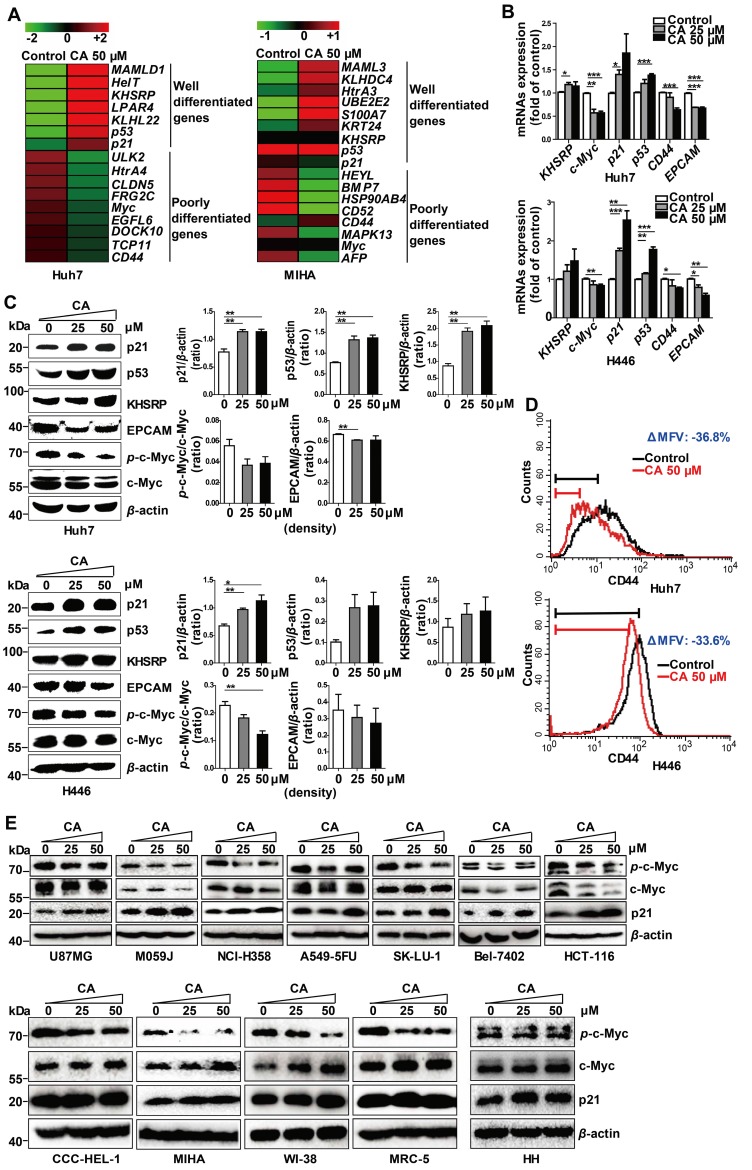
** CA regulated the expression of differentiation-related genes in Huh7 and H446 cells. (A)** mRNA expression of differentiation-related genes in Huh7 and MIHA cells treated with CA was analyzed by microarray and presented in the heat map, in which the red or green means relatively high or low gene expression, respectively. For Huh7 cells, CA up-regulated genes associated with differentiation, such as mastermind-like domain containing 1 (*MAMLD1*, fold change (FC) = +19.33), HEY-like transcription factor (*HelT*, FC = +9.92), K-homology splicing regulatory protein (*KHSRP*, FC = +7.01), lysophosphatidic acid receptor 4 (*LPAR4*, FC = +6.48), Kelch-like family member 22 (*KLHL22*, FC = +5.76), *p53* (FC = +4.01) and cyclin-dependent kinase inhibitor 1A (*CDKN1A*,* p21*, FC = +1.35); and it down-regulated genes associated with poor differentiation, such as Unc-51-like kinase 2 (*ULK2*, FC = -13.56), high-temperature requirement protein A4 (*HtrA4*, FC = -10.89), claudin 5 (*CLDN5*, FC = -10.53), FSHD region gene 2 family member C (*FRG2C*, FC = -9.48), V-myc avian myelocytomatosis viral oncogene homolog (*Myc*, FC = -6.86), EGF-like domain, multiple 6 (*EGFL6*, FC = -6.68), dedicator of cytokinesis 10 (*DOCK10*, FC = -5.36), T-complex 11 (*TCP11*, FC = -5.22) and *CD44* (FC = -1.50). The profile of MIHA cells was as comparison. **(B)** Change of mRNA expression in the microarray was verified by qRT-PCR assay both in Huh7 (upper) and H446 cells (lower). **(C)** Change of protein expression was confirmed by Western blotting in Huh7 (upper) and H446 cells (lower), and the results were normalized to that of *β*-actin. The density scanning was also performed. **(D)** Expression of CD44 in the Huh7 (upper) and H446 (lower) cells was evaluated by flow cytometry. To obtain the ΔMFV value, the mean fluorescence value (MFV) of the untreated control was subtracted by that of the MFV of CA treated group (50 μM). (E) Seven human solid tumor cell lines U87MG, M059J, NCI-H358, A549-5FU, SK-LU-1, Bel-7402, and HCT-116 (upper), 4 human non-cancer cell lines (CCC-HEL-1, MIHA, WI-38, and MRC-5), and primary human hepatocytes HH (lower) were treated with CA (25, 50 μM) for 24 h, followed by Western blotting for the protein expression of *p*-c-Myc, total c-Myc and p21. Data are presented as mean ± SEM. Three independent experiments were performed under identical condition. * p < 0.05, ** p < 0.01 and *** p < 0.001 (CA treated vs untreated control).

To understand the potential molecular mechanism(s) underlying the phenotypic change by CA, we investigated the differential mRNA expression. Huh7 and non-cancer liver MIHA cells were treated with 50 μM of CA for 24 h, followed by transcriptome microarray profiling. As shown in Figure 2A, CA increased expression of key genes in Huh7 cells associated with differentiation, for instance, *KHSRP*, *p53*, and* p21*. Simultaneously, CA treatment decreased the expression of genes associated with poor differentiation, such as c-*Myc* and *CD44,* among others. These differentiation-related genes were identified based on the gene function description in the Gene Bank database (https://www.ncbi.nlm.nih.gov/gene/) followed by literature review for confirmation. To validate the results, expression of the corresponding mRNAs and proteins of KHSRP, p53, p21, c-Myc, and CD44 was examined in Huh7 and H446 cells using qRT-PCR, Western blotting, or FCM analysis. The mRNA results were consistent with those from the transcriptome microarray (Figure 2B). Expression of p21, p53, and KHSRP proteins was significantly increased by CA, while phosphorylated c-Myc and total c-Myc protein expression dramatically decreased after CA treatment (25 or 50 μM) in both Huh7 and H446 cells (Figure 2C; right side: density scanning results). *EPCAM* is also a gene closely related to poor differentiation 33, and its expression was found to be decreased by CA in both cancer cell lines (Figures 2B-C), although its expression reduction in microarray was not substantial. CD44-positive cells declined by CA (50 μM) in Huh7 (ΔMFV = -36.8%) and H446 cells (ΔMFV = -33.6%) in the FCM analysis (Figure 2D). It appeared that CA caused a differentiation shift in cancer by regulating genes associated with cell differentiation.

We also examined seven other human solid tumor cell lines: U87MG (glioma), M059J (glioma), HCT-116 (colon carcinoma), NCI-H358 (non-small cell lung cancer), A549-5FU (lung cancer cells resistant to 5-FU), SK-LU-1 (lung adenocarcinoma), and Bel-7402 (hepatoma). Similar to the Huh7 and H446 cells, CA dose-dependently decreased *p*-c-Myc expression and increased p21 expression in all seven cell lines confirming the differentiation-inducing effect of CA in solid tumors (Figure 2E, upper; density scanning results, Figure S1A). Furthermore, four non-cancer human cell lines were tested for comparison, 2 liver cell lines (CCC-HEL-1 and MIHA) and two lung cell lines (WI-38 and MRC-5). As shown in Figure 2E (lower; for density scanning, see Figure S1B), in contrast to cancer cells, CA treatment showed no effect on p21 expression in these lines but decreased the phosphorylation of c-Myc to *p*-c-Myc in all four cell lines. In the microarray analysis with MIHA, CA regulated the expression of a set of differentiation-related genes (Figure 2A) different from those seen in the CA-treated Huh7 cells. The reason for the different response to CA between cancer and non-cancer cells remains unclear. Considering that the four non-cancer cell lines were isolated, immortalized *in vitro,* and cultivated in laboratories for years, their gene expression profiles might have shifted from their original normal patterns. Thus, primary human hepatocytes HH were examined. As shown in Figure 2E (lower) and Figure S1B, in contrast to the Huh7 cells following exposure to CA, HH cells showed almost no change in the expression in *p*-c-Myc, total c-Myc and p21. Thus, the cell differentiation response to CA appears to occur in the order of cancer cells > non-cancer immortalized cells > normal primary cells.

Since meristematic tissues are considered stem cells of plants, we induced human pluripotent stem (iPS) cells to examine the CA differentiation-inducing effect. We found that the mRNA expression of well-differentiated genes (like *p53*, *p21*) was significantly increased whereas that of poorly differentiated genes (like *c-Myc*, *EPCAM*, and* CD44*) declined in response to CA treatment (25 or 50 μM, 24 h) (Figure S2A). The results were confirmed by Western blotting (Figure S2B).

### CA elevated p21 expression by down-regulating the expression of the miR-17 family

**Figure 3:**
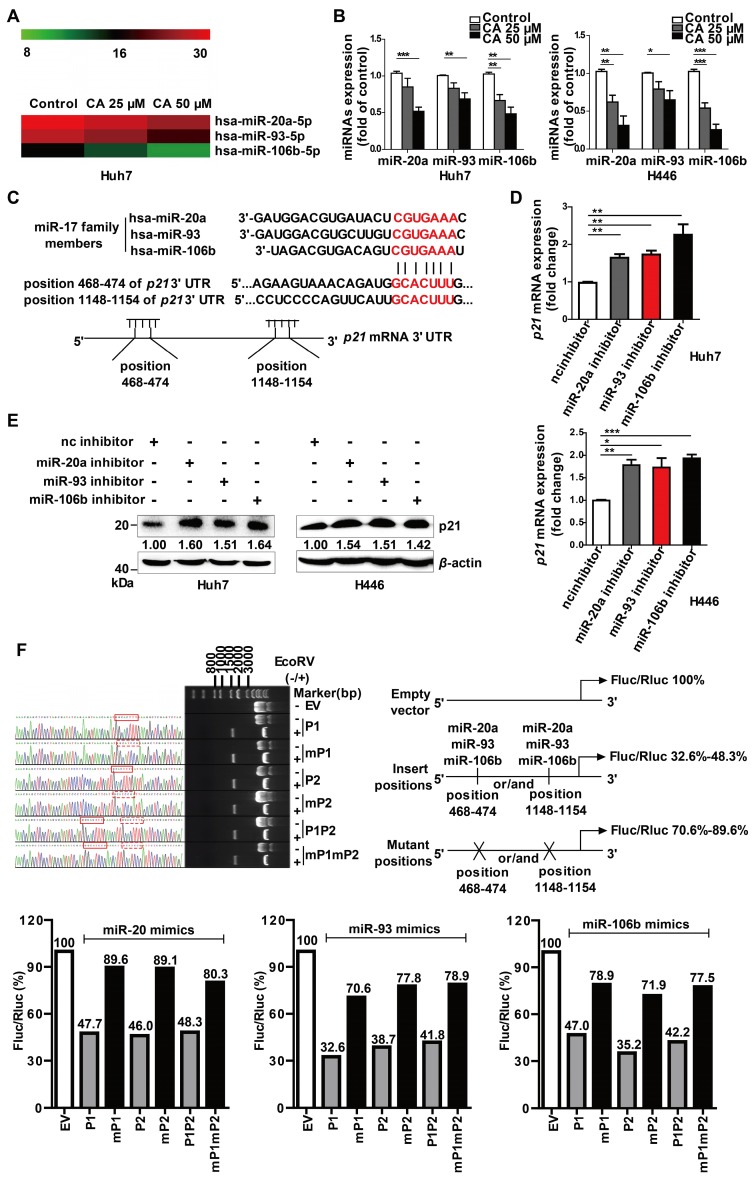
** P21 is the target gene of miR-20a, -93, and 106b, which were suppressed by CA. (A)** CA-caused expression change of the miR-17 family members is presented as the heat map, in which red or green means a relatively high or low expression of the genes, respectively; hsa,* Homo sapiens*. **(B)** Expression change of the miR-17 family members by CA in Huh7 (left) or H446 (right) cells was verified by qRT-PCR. Huh7 and H446 cells were treated with CA (25 or 50 μM for 24 h) followed by various measurements; data shown are mean ± SEM of 3 independent experiments (* p < 0.05, ** p < 0.01, *** p < 0.001, CA treated vs untreated). **(C)** Seed sequence of miR-17 family complemented to the *p21* mRNA 3' UTR was identified by using existing bio-information websites; the seed sequence of the miR-17 members 5'-AAAGUGC-3' and the complementary sequence in the *p21* mRNA 3' UTR are marked in red. **(D)** *p21* mRNA expression in Huh7 (left) and H446 (right) cells was assessed by RT- qPCR following treatment of cells with inhibitors of the miR-17 members; data shown are mean ± SEM of 3 independent experiments (* p < 0.05, ** p < 0.01 and *** p < 0.001, miRs inhibitor vs negative control inhibitor, nc inhibitor). **(E)** P21 protein expression in the Huh7 (left) and H446 (right) cells treated with miR-17 inhibitor by Western blotting. **(F)** P21 was identified as a target gene of miR-17 members, using dual luciferase reporter assay; three pairs of vectors were constructed, and the study fragments were obtained using specific primers, forward: 5'-GAAATTTGTGATGCTATTGC-3' and reverse: 5'-GCAATAGCATCACAAATTT-3', followed by sequencing. P1 represents a successful insertion into the oligonucleotide sequence of* p21* 3' UTR at position 468-474 (GCACTTT), the binding site of the miR-17 members (solid red line box), and mP1 is an insertion into the mutant sequence of *p21* 3' UTR at position 468-474 (GCA*TCC*, dashed red box). P2 represents an insertion into the oligonucleotide sequence of* p21* 3' UTR at position 1148-1154, the binding site of the miR-17 members, and mP2 is an insertion into the mutant sequences of *p21* 3' UTR at position 1148-1154 (GCA*TCC*). P1P2 are insertions into the oligonucleotide sequences of *p21* 3' UTR at both positions 468-474 and 1148-1154, the binding sites of miR-17 members, and mP1mP2 represent insertions into the mutant sequences of *p21* 3' UTR at positions 468-474 and 1148-1154 (GCA*TCC*). The data were normalized to EV as 100%. EV stands for empty vector.

As gene expression is largely controlled by the miRNA machinery, we next investigated whether CA regulated differentiation-related genes through miRNAs. The miRNA microarray profiling showed that the expression of the miR-17 family member's miR-20a, -93, and -106b was down-regulated in a dose-dependent manner after exposure of the cells to CA (25 or 50 μM for 24 h) (Figure 3A). The results were then confirmed in both Huh7 and H446 cells by the qRT-PCR assay (Figure 3B). The expression of the 3 miR-17 members was also decreased by CA (25 or 50 μM for 24 h) in iPS cells (Figure S2C). Subsequently, we analyzed three miRNA-related databases, the MiRNA.org (www.microrna.org), TargetScan Human (www.targetscan.org) and PicTar (pictar.mdc-berlin.de), to identify the potential target genes of miR-20a, -93, and -106b. We found that the *p21* 3' UTR (untranslated region) sequence had two sites (with a sequence of 5'-GCACUUU-3'), position 468-474 and position 1148-1154, which matched the seed sequence (5'-AAAGUGC-3') of the miR-17 family (Figure 3C), suggesting that *p21* is a possible target gene of the miR-17 family members. We next investigated whether p21 expression was influenced after diminution of the three miR-17 family members. As shown in Figure 3D, transfection of Huh7 or H446 cells with the miR-20a, -93, or -106b specific inhibitor (single-chain nucleotide complementary to the three miRNAs mature sequence) increased *p21* mRNA expression. Consistent with this observation, elevated p21 protein was detected in the Western blotting assay (Figure 3E). The dual luciferase reporter assay demonstrated that overexpression of miR-20a, miR-93, or miR-106b mimics (50 nM) reduced the luciferase activity of the vectors (200 ng) with the *p21* 3' UTR binding sites. The ratio of Firefly to Renilla luciferase activity (Fluc/Rluc) ranged from 32.6%-48.3% of that of the empty vector control. In contrast, overexpression of the miR-20a, miR-93, or miR-106b mimics (50 nM) in cells with vectors carrying mutated* p21* 3' UTR binding sites (200 ng) showed minor effects on luciferase activity, and the Fluc/Rluc value ranged from 70.6%-89.6% of the control (Figure 3F, scheme). The results indicated that the two binding sites in the *p21* 3' UTR sequence were the responsive elements for the three miR-17 family members.

Also, transfection of the CA-treated Huh7 or H446 cells with mimics of miR-20a, miR-93, miR-106b or p21-siRNAs (si-p21) eliminated the p21 up-regulatory effect of CA and increased c-Myc and CD44 expression (Figure S3). As p21 has a positive role in cell differentiation, in contrast to that of c-Myc and CD44, it appeared that through down-regulation of miR-20a, -93, and -106b expression, CA stabilized the 3' UTR of *p21* mRNA, increased the intracellular level of p21 protein, and triggered the differentiation cascade in tumor cells.

### CA-caused c-Myc SUMOylation resulted in miR-17 family decline and p21 elevation

**Figure 4:**
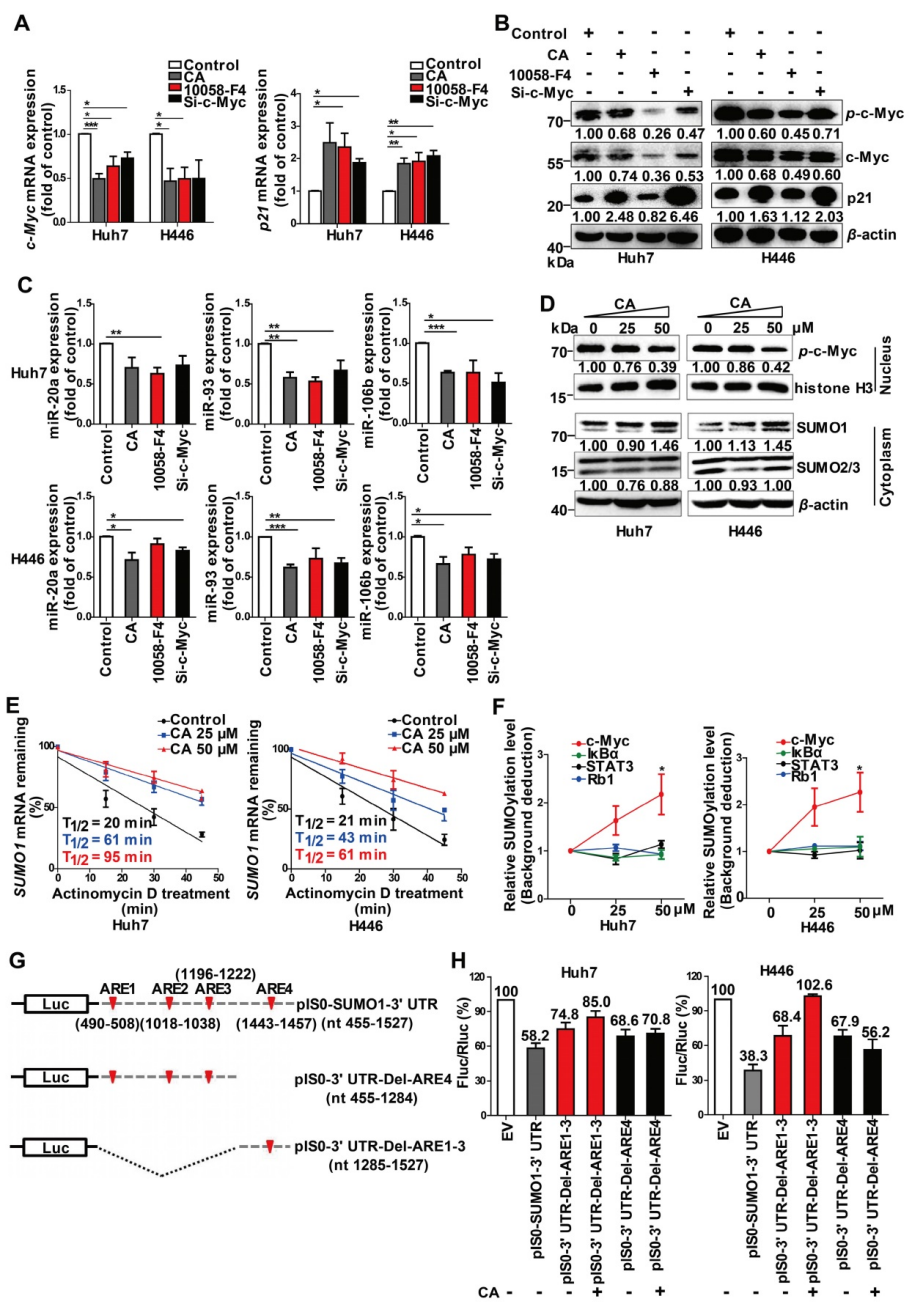
** SUMOylation of c-Myc by CA decreased *p*-c-Myc and miR-17 family and increased p21.** Huh7 and H446 cells were treated with CA (50 μM), or 10058-F4 (30 μM, c-Myc-specific inhibitor), or c-Myc siRNA (50 nM), respectively, for 24 h. **(A)** Expression of *c-Myc* (left) and *p21* (right) mRNAs by qRT-PCR assay. **(B)** Protein expression of *p*-c-Myc, total c-Myc, and p21 in Huh7 (left) and H446 (right) cells by Western blotting; the results were normalized to *β*-actin in density scanning with the control group as 1. **(C)** Levels of the miR-17 members in Huh7 (upper) and H446 (lower) cells, treated with CA or 10058-F4 or c-Myc siRNA at conditions mentioned above, assessed by qRT-PCR. **(D)** Expression of nuclear protein *p*-c-Myc in Huh7 (left) and H446 (right) cells by Western blotting and normalized to histone H3 with density scanning (upper); Cytoplasmic proteins SUMO1 and SUMO2/3 in Huh7 and H446 cells determined by Western blotting and normalized to *β*-actin (lower), with the control (0 μM) as 1. **(E)** CA increased *SUMO1* mRNA stability. Huh7 (left) and H446 (right) cells were treated or untreated with CA for 12 h. Subsequently, actinomycin D (5 μM) was added at indicated time points. mRNA degradation was analyzed by qRT-PCR. Decay curves were plotted versus time. **(F)** Sumoylation of c-Myc, I*κ*B*α*, Rb1, and STAT3 in Huh7 (left) and H446 (right) cells treated with CA (25, 50 μM,) was analyzed using the EpiQuik^TM^ Protein Sumoylation Assay Kit (Epigentek). **(G)** Schematic presentation of the *SUMO1* mRNA 3' UTR inserted into pIS0 vector and construction of Luc-*SUMO1* mRNA 3' UTR fusions. **(H)** ARE-4 region involved in the stabilizing effect of CA on *SUMO1* mRNA, using dual luciferase reporter assay. Data were normalized to EV as 100%. EV stands for empty vector of pIS0. All data are presented as mean ± SEM of 3 independent experiments. * p < 0.05, ** p < 0.01 and *** p < 0.001 (treated vs untreated control).

c-Myc is known to be an important regulator for miRNAs 34 and might play a role in the expression of miR-17 family members. Thus, we used a c-Myc specific inhibitor, 10058-F4, as well as c-Myc siRNAs in the *in vitro* experiments. Treatment with CA (50 μM), 10058-F4 (30 μM) or si-c-Myc (50 nM) decreased *c-Myc* mRNA expression in Huh7 and H446 cells (Figure 4A, left) with a simultaneous increase in the expression of *p21* mRNA (Figure 4A, right), showing the negative relationship between c-Myc and p21. The results were confirmed at the protein level by Western blotting in which CA showed an advantage in elevating p21 protein expression superior to that of 10058-F4 (Figure 4B). Moreover, down-regulated expression of miR-20a, miR-93, and miR-106b (Figure 4C) was also observed in both tumor cell lines treated with CA or with the c-Myc inhibitors. This finding suggested that the down-regulation of c-Myc by CA might up-regulate p21 expression through abrogating the expression of miR-17 family members.

We also detected an increase in the small ubiquitin-like modifier 1 (SUMO1) protein expression but not SUMO2 and SUMO3 proteins in the Huh7 and H446 cells cytoplasm following CA treatment (Figure 4D, lower). It significantly promoted the SUMO1 in the cytoplasm and simultaneously decreased the levels of *p*-c-Myc (Figure 4D, upper) in the cell nucleus. CA (50 µM) increased the stability of the* SUMO1* mRNA transcripts in Huh7 and H446 cells with an increase in T_1/2_ by 3.75- or 1.9-fold, respectively (Figure 4E). The CA-caused SUMOylation of c-Myc was dose-dependent and selective as it was negative for other proteins such as I*κ*B*α*, Rb1 and STAT3 (Figure 4F). It appeared that CA stabilized the *SUMO1* mRNA, increased the intracellular SUMO1 protein, selectively promoted sumoylation of c-Myc instead of phosphorylation, and thus decreased the expression of the miR-17 family members 34, leading to an elevation of p21 protein in tumor cells.

We then assessed which regulatory sequences in the SUMO1 mRNA 3' UTR region mediated the effect of CA on *SUMO1* mRNA stability. The SUMO1 mRNA 3' UTR sequence contains 4 AU-rich elements (ARE1-4) with a total length of 1073 bp.

We selected two consecutive regions containing ARE1-3 (nt 490-1222) and ARE4 (nt 1443-1457), which were then individually inserted into the pIS0 vector (Figure 4G). The cells were transfected with pIS0, pIS0-SUMO1-3' UTR (nt 455-1527), pIS0-SUMO1-3' UTR-Del-ARE4 (nt 455-1284), or pIS0-SUMO1-3' UTR-Del-ARE1-3 (nt 1285-1527). Transfection of the cells with the vectors containing the full-length 3' UTR, ARE1-3 or ARE4 reduced the luciferase activities of the pIS0 vectors (EV) to various degrees in both Huh7 and H446 cells (Figure 4H). Treatment of the transfected cells with CA (50 µM, for 20 hr) attenuated the reduction effect caused by the ARE4-containing vectors but not those containing ARE1-3- suggesting that the ARE4 region (nt 1285-1527) might be the main responsive element for CA-induced stabilization of SUMO1 (Figure 4H).

### CA induced differentiation in mouse xenograft tumors

**Figure 5:**
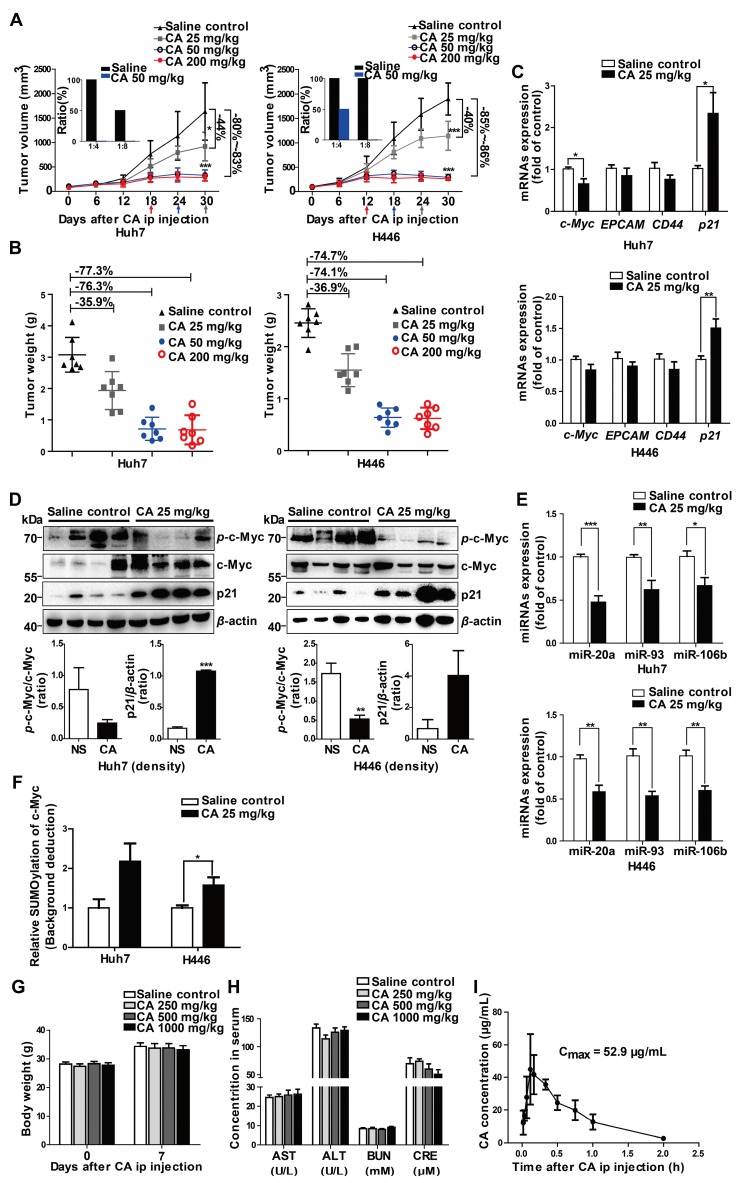
** CA terminated Huh7 and H446 tumor growth *in vivo.* (A)** Tumor growth inhibition by CA (ip, 25 or 50 or 200 mg/kg/d) in NOD/SCID mice inoculated with Huh7 (left) or H446 (right) cells. Arrows indicate the xenograft tumor entered non-growth phase, after treatment with CA at different doses. Tumor tissues taken from the CA-treated mice largely lost tumorigenicity after re-implantation of the tumor cells into naïve mice for 28 days (inserts, n = 4). Data are presented as mean ± SD. * p < 0.05 and *** p < 0.001 (CA treated versus saline control). **(B)** Tumor weight in the NOD/SCID mice inoculated with Huh7 (left) or H446 (right) cells followed by CA treatment (ip, 25 or 50 or 200 mg/kg/d) and saline treatment used as a control. CA treatment (ip, 25 mg/kg/d or 50 mg/kg/d or 200 mg/kg/d) yielded 35.91%, 76.34% or 77.33% tumor weight inhibition of Huh7 tumors and 36.90%, 74.14% or 74.72% tumor weight reduction of H446 tumors, respectively. **(C)** mRNA expression of the study genes in Huh7 (upper) or H446 (lower) tumor tissues taken from the mice treated with CA (25 mg/kg/d) was examined by qRT-PCR (n = 6). **(D)** Protein expression of c-Myc and p21 in Huh7 (left) or H446 (right) tumors was analyzed by Western blotting (n = 6, upper panel). Density scanning results are presented as well (lower panel). **(E)** Expression levels of miR-17 family members in Huh7 (upper) or H446 (lower) tumors taken from the CA-treated mice was assessed by qRT-PCR (n = 6). **(F)** The nucleus sumoylation of c-Myc in Huh7 or H446 tumors was measured using EpiQuik^TM^ Protein Sumoylation Assay Kit (n = 6). **(G)** Body weight measurement on day-7 after CA injection (ip; 1000, or 500, or 250 mg/kg/d; n = 10 for each group). **(H)** Liver and kidney function remained normal after CA treatment. AST: aspartate transaminase (U/L); ALT: alanine aminotransferase (U/L); BUN: blood urea nitrogen (mM); CRE: creatine (μM). The data are presented as mean ± SEM. * p < 0.05, ** p < 0.01 and *** p < 0.001 (CA treated versus normal saline control). **(I)** C_max_ of CA in rats was measured after ip injection of CA (75 mg/kg). Three rats were used for each time point. C_max_, the maximum concentration in blood. Data are presented as mean ± SD.

To investigate the differentiation-based therapeutic effect of CA *in vivo*, we used the Huh7 hepatoma and H446 lung cancer xenograft models in the NOD/SCID mice. CA treatment with ip administration of 25 mg/kg/d, 50 mg/kg/d, or 200 mg/kg/d slowed down the growth of the tumor mass, yielding 44.0%, 80.1% or 83.1% tumor volume inhibition of Huh7 tumors and 39.9%, 84.6%, or 86.3% tumor reduction of H446 tumors, respectively (Figure 5A). Accordingly, tumor weight was declined by CA as well (Figure 5B). Increasing the CA dosage up to 200 mg/kg/d (ip) did not cause further decline of the tumor mass but accelerated the entry of the xenograft tumor into the non-growth plateau (Figure 5A). No animal toxicity was observed. As both the Huh7 and H446 tumors maintained non-growth after CA treatment (50 or 200 mg/kg/d) for 12 or 18 days (Figure 5A), we assumed that, similar to the iPS cells, most of the remaining tumor cells, including tumor stem cells, might have been differentiated by CA. By analyzing differentiation-related genes, we found that *c-Myc*, *EPCAM*, and *CD44* mRNA levels decreased in both Huh7 and H446 (Figure 5C) xenograft tumors in the mice treated with CA (ip, 25 mg/kg/d), while the *p21* mRNA level increased (Figure 5C) compared with the saline-treated control. Western blotting showed that CA treatment reduced the level of *p*-c-Myc protein but elevated the p21 protein level in the tumor tissue (Figure 5D). Furthermore, the expression of miR-20a, miR-93, and miR-106b was down-regulated both in Huh7 and H446 (Figure 5E) xenografts in mice treated with CA (ip, 25 mg/kg/d). Sumoylation of c-Myc was also increased after the CA treatment (Figure 5F). The results obtained for CA-induced solid tumor differentiation *in vivo* were consistent with those observed *in vitro*.

Next, we digested and homogenized 25 mg of the tumor tissue pooled from the saline control mice (n = 5) or from the CA-treated (50 mg/kg/d) mice (n = 5), followed by reimplantation of the tumor cell suspension (at 1:4 and 1:8 dilutions) into naïve SCID mice (n = 4 for each group). As displayed in Figure 5A (inserts), on day 28 after re-implantation, no tumors developed in the mice inoculated with Huh7 tumor cells collected from the CA-treated mice (both 1:8 and 1:4 dilutions). On the other hand, there was tumor development in mice implanted with saline-treated controls with all mice tumor-positive when implanted with cells at a 1:4 dilution and half of the mice growing tumors at a 1:8 dilution. For the H446 lung cancer xenografts, while all mice inoculated with the tumor suspension from saline-treated H446 tumor-bearing mice developed tumors (for both 1:4 and 1:8 dilutions), the mice implanted with a 1:8 dilution originating from xenografts of mice treated with CA did not develop tumors; but half of them developed tumors at a 1:4 dilution. These results suggested that CA might decrease tumorigenicity through differentiating cancer cells in the tumor mass.

The safety test showed that all mice survived for 7 days after single dosing of CA ranging from 250 to 1000 mg/kg (ip). Compared with the saline control group, CA ip injection caused no change in body weight (Figure 5G) and no damage to the liver and kidney function, with serum AST, ALT, BUN and CRE levels within the normal range (Figure 5H). These observations were consistent with excellent biocompatibility and safety of CA in humans. When the blood concentration of CA (ip, 75 mg/kg) was examined in the SD rats (3 rats per time point), the average C_max_ of CA in the ip injection was 52.9 µg/mL (147 µM), which was higher than the drug concentration used in the *in vitro* experiments (Figure 5I).

### CA was effective for glioma cells *in vitro* and in animal models

Besides liver and lung cancer, we also investigated the efficacy of CA in glioma cells. As is evident from Figure 2E, human glioma cells were sensitive to CA, showing reduced *p*-c-Myc and elevated p21 expression similar to the results observed in hepatoma, lung cancer as well as colon cancer. Further investigation of CA in gliomas was designed to identify specific biomarkers or indication of differentiation in brain tumors. Toward this end, the cellular expression of Tuj1 and glial fibrillary acidic protein (GFAP) in glioma cells was assessed as signature markers for neurons and astrocytes, respectively 35, 36.

**Figure 6:**
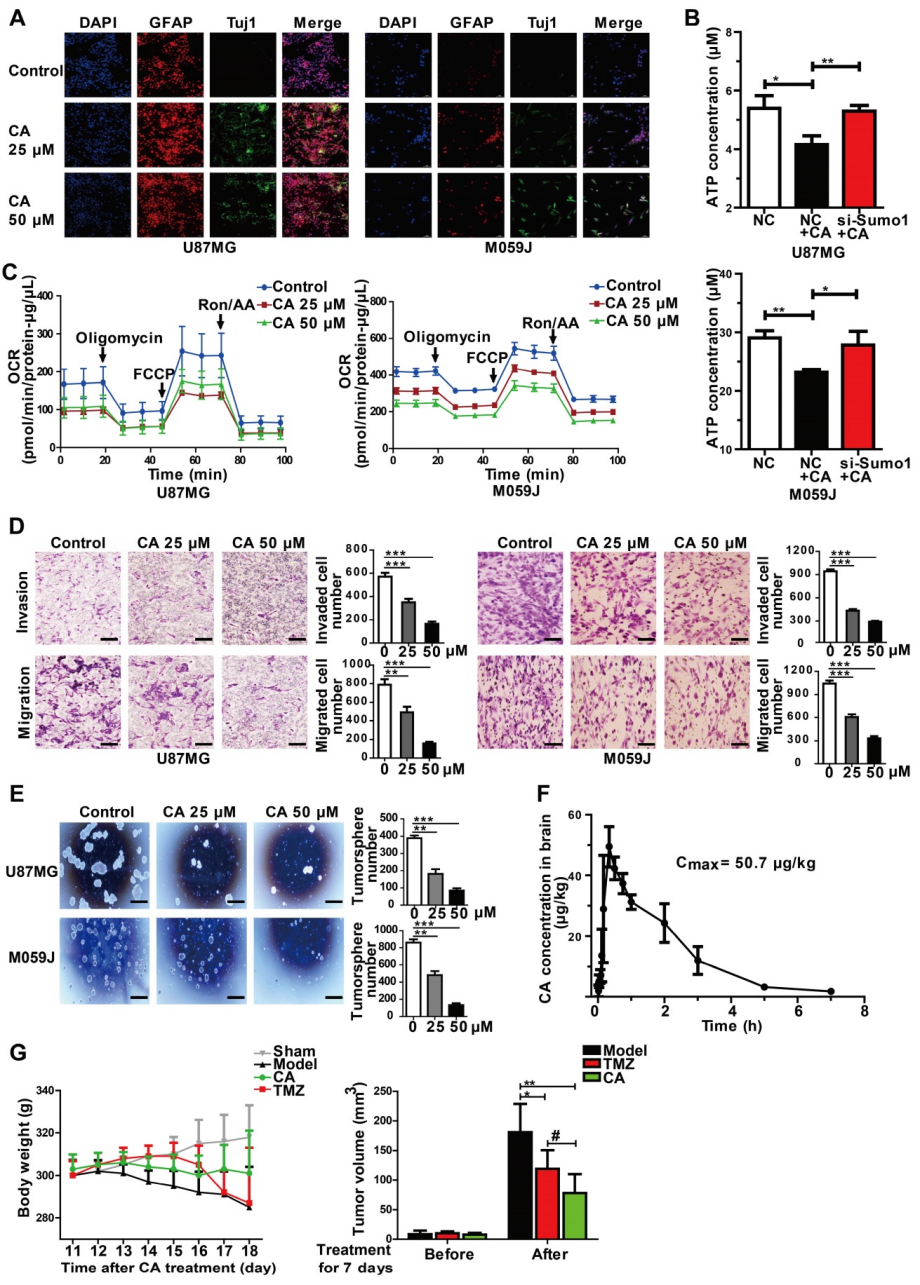
** CA is effective for glioma cells *in vitro* and *in vivo.* (A)** Immunofluorescence assay was shown the expression of glioma cell differentiation markers, GFAP (the astrocyte marker) and Tuj1 (the neuron marker) in U87MG and M059J cells after exposure to CA (Bar = 50 μm). **(B)** Total ATP level was detected by measuring the luminescence in U87MG and M059J cells after respective treatment. **(C)** Mitochondrial stress test of U87MG and M059J cells after exposure to CA. Oxygen consumption rate (OCR) was calculated by the Seahorse XF-24 analysis. **(D)** Representative images of invasion (upper) and migration (lower) of U87MG and M059J cells after exposure to CA. Data are presented as mean ± SEM, ** p < 0.01, *** p < 0.001 (CA treated versus untreated control. Scale bars indicate 10 μm). **(E)** Glioma cell sphere formation of U87MG (upper) and M059J (lower) cells after exposure to CA stained with 0.4% Trypan blue, calculated and observed at 200 × magnifications under the microscope. Data are presented as mean ± SEM, ** p < 0.01, *** p < 0.001 (CA treated versus untreated control. Scale bars indicate 10 μm). **(F)** CA concentration in the brain was measured at consecutive time points post injection (ip) with 3 rats for each time point. **(G)** CA treatment (75 mg/kg, ip, bid, for 7 days) did not significantly reduce the body weight of the rats as compared to the sham group (left), and inhibited the C6 glioma tumor growth in rats with a higher inhibitory efficacy than that of temozolomide (right; 20 mg/kg/d, oral, for 5 days). Data are presented as mean ± SD, ^#^ p < 0.05, CA group versus TMZ group; * p < 0.05, ** p < 0.01, CA or TMZ group versus model group; n = 6 for each group).

Immunofluorescent staining showed increased expression of Tuj1 and GFAP both in the U87MG and M059J glioma cells after CA treatment (25 or 50 μM, 24 h) indicating the shift toward neurological characteristics (Figure 6A). Since cancer cells exhibit increased glucose utilization and energy metabolism 5, 37, mitochondrial function was examined in glioma cells with or without CA treatment. As shown in Figure 6B, CA treatment decreased energy metabolism in mitochondria indicating reduced ATP production in the U87MG and M059J glioma cells by about 21%. Also, silencing SUMO1 expression by transfection of SUMO1-specific siRNA abolished the effect of CA on ATP reduction suggesting a SUMO1-dependent action of CA on mitochondria (Figure 6B). Thus, it appears that SUMO1 is critical for the mechanism of action of CA. Oxygen consumption rate (OCR, both basal or maximal) in the two glioma cell lines was reduced after treating the cells with CA (25 μM or 50 μM, 24 h; Figure 6C), indicating inhibition of oxidative phosphorylation in the mitochondrial respiratory chain by CA. Accordingly, the cell mobility assay showed that the number of invading (Figure 6D, upper) and migrating glioma cells (Figure 6D, lower) was significantly reduced in the M059J or U87MJ cells treated with CA (25 or 50 µM for 24 h) as compared to the untreated ones possibly due to the decline in the ATP level. Finally, in the neurosphere culture, we observed inhibition of the sphere formation by CA both in U87MG and M059J cells (Figure 6E) indicative of decreased malignancy in the CA-treated glioma cells 38, 39. These changes of differentiation markers and cell behavior in glioma strongly suggest maturation toward differentiated cancer cells.

The pharmacokinetic study in rats revealed that CA was able to cross the blood-brain barrier and enter the brain tissue 10-20 min after ip injection (75 mg/kg, Figure 6F). The maximum brain concentration of CA in the rats was 50.7 µg/kg (n = 3). CA administration (75 mg/kg, ip, bid) caused significant inhibition of tumor growth of the C6 glioma in rats (** p < 0.01, n = 6 for each group, Figure 6G, right) with an inhibition rate of 57% of the tumor volume by MRI. The CA anticancer efficacy was comparable to that of temozolomide (TMZ; ^#^ p < 0.05, CA group vs TMZ group), the first-line drug for glioma in the clinic (Figure 6G, ref. 40). Continuous injection of CA showed no toxicity in the rats as evaluated by the rat body weight measurements (Figure 6G, left). The sham group (n = 6) showed no tumors in the brain based on MRI detection (data not shown).

## Discussion

The present study showed, for the first time, that CA induced differentiation in solid tumors and inhibited cancer cell proliferation, mobility, invasion, ATP production in mitochondria, and tumor clone formation. These changes were accompanied by normalization in morphology as well as altered gene expression in cancer cells, favoring a shift toward differentiation. Mechanistic studies revealed that CA increased SUMO1 protein expression by stabilizing *SUMO1* mRNA, thus causing SUMOylation of c-Myc and decreasing *p*-c-Myc, which in turn downregulated the expression of the three miR-17 family members. The decrease in miR-20a, miR-93 and miR-106b levels promoted p21 expression leading to cell differentiation. Activation of the “c-Myc SUMOylation/miR-17 reduction/p21 elevation axis” by CA resulted in cell differentiation and attenuated the cancer behavior of solid tumor cells.

Our study showed that the SUMOylation of c-Myc, down-regulated miR-17 family members and up-regulated p21 are important steps for CA-mediated differentiation of cancer cells. SUMOylation is a posttranslational modification that represents a process of protein conjugation with SUMO proteins 1-4. SUMO1, SUMO2, and SUMO3 are rich in vertebrate tissues, and SUMO4 is expressed only in the kidney, lymph nodes and spleen 41. SUMOylation controls cell cycle progression and embryo development 42, 43. Functional proteins, such as MDM2, Rb, and STAT3, among others, can be modified by SUMOs 44-46. Of these SUMO proteins, SUMO2-mediated SUMOylation plays an important role in ATO-induced differentiation therapy 47. The c-Myc protein contains 10 lysine residues that can be conjugated with the SUMO protein to modify its oncogenic activity 48. Herein, we showed that CA increased SUMO1 expression and boosted c-Myc SUMOylation. The SUMOylation at the lysine sites might protect c-Myc from phosphorylation at the S62 and/or T58 sites in its C-terminus 49, 50. The decrease in *p*-c-Myc in response to CA suppressed the transcriptional activity of the miR-17 family consistent with previous reports 51, 52. For mitochondria, increased expression of SUMO1 reduced ROS production probably through its action on NADPH oxidases 53. Our results showed that the declined ATP production by CA was SUMO1-dependent, demonstrating, for the first time, the role of SUMO1 in the oxidative phosphorylation for ATP production. The relationship between CA-caused ATP reduction and SUMO1 function in mitochondria needs to be further investigated.

In the present study, we discovered that CA acts as a suppressor for three members of the miR-17 family. It is well recognized that miRNAs can act as oncomiRs or antioncomiRs in cancers 54-57. Numerous miRs have been reported to participate in cell cycle regulation, drug resistance, tumor differentiation, or cell “stemness” 58-61. As miR-20a, miR-93, and miR-106b share the same critical seed sequence, they are assigned to the miR-17 family (http://www.mirbase.org/). It has been reported that the upregulation of miR-20a, miR-93 and miR-106b is associated with a poor prognosis in breast cancer, pediatric glioma and hepatocellular carcinoma and is linked to the promotion of tumor proliferation and invasion 62-65. In the present study, we identified miR-17 family members as oncomiRs that function by inhibiting p21.

Recently, we have been interested in meristematic tissues in plants in search of effective, but non-toxic, tumor differentiation inducers. In this context, CA has been shown to stimulate a complex signaling network that causes phenotypic transformation toward cell differentiation. A decrease in gene expression in response to CA was observed in a group of genes associated with poor differentiation. Among these genes, *CD44* and *EPCAM* are known to be highly expressed in progenitor cells as well as in undifferentiated stem cells 33, 66. Of the oncogenes affected by CA, c-Myc plays an important role in malignant progression by activating a variety of genes (such as those encoding CDKs) and promotes the expression of oncomiRs. For instance, there are four c-Myc E-box binding sites in the CDK4 promoter region, which elevate CDK4 expression and play a critical role in cell-cycle progression by mediating its oncogenic function 67. Furthermore, c-Myc drives the metabolism of glucose and glutamine for the production of ATP needed for cell proliferation 68. High expression of c-Myc promotes the expression of multi-oncomiRs, such as miR-17-5p, -92, -106b, -19a, -19b, and miR-20a 69. Other genes, which likely promote differentiation and show increased expression levels by CA, include *MAMLD1*, *KHSRP*, *LPAR4*, *KLHL22*, *p53*, and *p21* 70-77. For example, p21 is well-recognized as an inhibitor of CDKs and acts at the G_1_/S phase checkpoint to determine the cell fate 78.

Human p21 is an important cellular component that responds to cellular stress and DNA damage 79. It acts on multiple pathways, leading to cell growth suspension, differentiation, or cellular senescence 80. For example, p21 prevents retinoblastoma protein (pRb) from undergoing phosphorylation 81 and increases the E2F-associated phosphor-protein (EAPP) level to maintain gene regulation by E2F transcription factors in a repressed status and to induce cell cycle arrest 82. P21 also interacts with proliferating cell nuclear antigen (PCNA), which is linked to DNA metabolism and repair in response to radiation damage 83. As p21 is widely accepted as a positive factor for differentiation or cellular senescence, we used it as one of the important differentiation indicators in the study. Using bioinformatics and the dual luciferase reporter assay, we discovered that the miR-17 family members were able to bind the 3' UTR sequence in *p21* through complementary base pairing and down-regulated p21 expression. Thus, the suppression of miR-17 family members by CA resulted in increased expression of p21, which then acted as a tumor suppressor protein to induce cell cycle arrest at the G_1_/S checkpoint and tumor differentiation. However, the involvement of other pathways could not be excluded, as cell differentiation represents a complex process that requires the synchronized operation of several genes/proteins.

In animal experiments, we showed that CA treatment terminated tumor growth. The detection of the genes/proteins associated with differentiation in tumors from the CA-treated animals revealed a profile identical to that observed *in vitro*. Through tumor re-implantation, we showed that CA also differentiated cancer stem cells. This inference was based upon the observation that the residual tumor cells from the CA-treated animals largely lost the ability to grow new tumors *in vivo*. It is of note that the tumor inhibition effect of CA in glioma was not as good as that in hepatoma or lung cancer, probably because of the low CA concentration in the brain. The mouse experiments showed a CA concentration in brain (the original CA compound) lower than that used in cell culture. The possible explanations for the CA's effect could be at least 1) CA has 6 structure-similar metabolites *in vivo* which might also be active against cancer 84, and 2) CA is a substrate of P-glycoprotein (P-gp) 85, 86; thus, the *in vivo* metabolites of CA might increase CA intracellular concentration by blocking P-gp efflux competitively, forming a synergistic action against glioma.

The CA-induced differentiation response in cancer cells was not detectable in primary human cells, probably because they are already differentiated cells. In animal experiments, CA terminated tumor growth by inducing differentiation and most of the tumor tissue taken from the CA-treated mice was no longer tumorigenic. More significantly, ip administration of CA exhibited no toxicity even at high doses. More research is needed to elucidate the molecular details of the differentiation of cancer cells induced by CA.

Taken together, we found that CA functioned as a safe differentiation inducer for solid tumors. After observing the exceptional therapeutic effect and excellent safety by CA in the recurrent high-grade glioma patients in the Phase I study (NCT02728349), CA has been approved by China FDA for Phase II trial (NCT03758014). Our study strongly suggested that “educating” cancer cells, rather than killing them, is a good option for future anticancer therapy. We consider CA as a promising therapeutic agent for cancer.

## Supplementary Material

Supplementary figures and tables.

## Abbreviations

CA: chlorogenic acid
DMSO: dimethyl sulfoxide
ATO: arsenic trioxide
ATRA: all-trans retinoic acid
ROS: reactive oxygen species
CFDA: China food and drug administration
OCR: oxygen consumption rate
TMZ: temozolomide
pRb: retinoblastoma protein
EAPP: E2F-associated phosphor-protein
PCNA: proliferating cell nuclear antigen
P-gp: P-glycoprotein
Td: doubling-time
miRNAs: microRNAs
SUMO1: small ubiquitin-like modifier 1
qRT-PCR: quantitative real-time PCR
AREs: AU-rich elements
FCCP: carbonyl cyanide p-trifluoro-methoxyphenyl hydrazine
Ron/AA: rotenone plus antimiycin A
TIR: tumor inhibition rate
TV: tumor volume

## References

[1] Cantone I, Fisher AG (2013). Epigenetic programming and reprogramming during development. Nat Struct Mol Biol.

[2] Chen CZ, Li L, Lodish HF, Bartel DP (2004). MicroRNAs modulate hematopoietic lineage differentiation. Science.

[3] Lazebnik Y (2010). What are the hallmarks of cancer?. Nat Rev Cancer.

[4] McAvoy JW, Chamberlain CG (1989). Fibroblast growth factor (FGF) induces different responses in lens epithelial cells depending on its concentration. Development.

[5] Zeng F, Chen X, Cui W, Wen W, Lu F, Sun X (2018). RIPK1 binds MCU to mediate induction of mitochondrial Ca^2+^ uptake and promotes colorectal oncogenesis. Cancer Res.

[6] Pályi I (1988). Heterogeneity of the response to inducers of differentiation and to cytostatics of tumor cell populations. Pathol Res Pract.

[7] Zhang LP, Jiang JK, Tam JW, Zhang Y, Liu XS, Xu XR (2001). Effects of Matrine on proliferation and differentiation in K-562 cells. Leuk Res.

[8] Huang ME, Ye YC, Chen SR, Chai JR, Lu JX, Zhao L (1988). Use of all-trans retinoic acid in the treatment of acute promyelocytic leukemia. Blood.

[9] Warrell RP Jr, Frankel SR, Miller WH Jr, Scheinberg DA, Itri LM, Hittelman WN (1991). Differentiation therapy of acute promyelocytic leukemia with tretinoin (all-trans-retinoic acid). N Engl J Med.

[10] Ma H, Yang J (2015). Insights into the all-trans-retinoic acid and arsenic trioxide combination treatment for acute promyelocytic leukemia: a meta-analysis. Acta Haematol.

[11] Shepshelovich D, Oniashvili N, Parnes D, Klein A, Muchtar E, Yeshaya J (2015). Acute promyelocytic leukemia with isochromosome 17q and cryptic PML-RARA successfully treated with all-trans retinoic acid and arsenic trioxide. Cancer Genet.

[12] Zhang XW, Yan XJ, Zhou ZR, Yang FF, Wu ZY, Sun HB (2010). Arsenic trioxide controls the fate of the PML-RARalpha oncoprotein by directly binding PML. Science.

[13] Emadi A, Gore SD (2010). Arsenic trioxide - an old drug rediscovered. Blood Rev.

[14] Doonan JH, Sablowski R (2010). Walls around tumours - why plants do not develop cancer. Nat Rev Cancer.

[15] Freeling M (1992). A conceptual framework for maize leaf development. Dev Biol.

[16] Koroleva OA, Gibson TM, Cramer R, Stain C (2010). Glucosinolate-accumulating S-cells in arabidopsis leaves and flower stalks undergo programmed cell death at early stages of differentiation. Plant J.

[17] Vanyushin BF, Ashapkin VV, Aleksandrushkina NI (2017). Regulatory peptides in plants. Biochemistry (Mosc).

[18] Marhava P, Bassukas AEL, Zourelidou M, Kolb M, Moret B, Fastner A (2018). A molecular rheostat adjusts auxin flux to promote root protophloem differentiation. Nature.

[19] Benjamins R, Scheres B (2008). Auxin: the looping star in plant development. Annu Rev Plant Biol.

[20] Fenech M, Amaya I, Valpuesta V, Botella MA (2019). Vitamin C content in fruits: biosynthesis and regulation. Front Plant Sci.

[21] Schmidt R, Schippers JH (2015). ROS-mediated redox signaling during cell differentiation in plants. Biochim Biophys Acta.

[22] Spoel SH, Dong X (2012). How do plants achieve immunity? Defence without specialized immune cells. Nat Rev Immunol.

[23] Zhang B, Chen N, Chen H, Wang Z, Zheng Q (2012). The critical role of redox homeostasis in shikonin-induced HL-60 cell differentiation via unique modulation of the Nrf2/ARE pathway. Oxid Med Cell Longev.

[24] Zhang TD, Chen GQ, Wang ZG, Wang ZY, Chen SJ, Chen Z (2001). Arsenic trioxide, a therapeutic agent for APL. Oncogene.

[25] Honma Y (2002). Cotylenin A-a plant growth regulator as a differentiation-inducing agent against myeloid leukemia. Leuk Lymphoma.

[26] Yang H, Yuan B, Li L, Chen H, Li F (2004). HPLC determination and pharmacokinetics of chlorogenic acid in rabbit plasma after an oral dose of Flos Lonicerae extract. J Chromatogr Sci.

[27] Watanabe T, Arai Y, Mitsui Y, Kusaura T, Okawa W, Kajihara Y (2006). The blood pressure-lowering effect and safety of chlorogenic acid from green coffee bean extract in essential hypertension. Clin Exp Hypertens.

[28] Yonathan M, Asres K, Assefa A, Bucar F (2006). In vivo anti-inflammatory and anti-nociceptive activities of cheilanthes farinosa. J Ethnopharmacol.

[29] Liang N, Kitts DD (2015). Role of chlorogenic acids in controlling oxidative and inflammatory stress conditions. Nutrients.

[30] Park SH, Baek SI, Yun J, Lee S, Yoon DY, Jung JK (2015). IRAK4 as a molecular target in the amelioration of innate immunity-related endotoxic shock and acute liver injury by chlorogenic acid. J Immunol.

[31] Nakashima H, Tsujimura K, Irie K, Ishizu M, Pan M, Kameda T (2018). Canonical TGF-β signaling negatively regulates neuronal morphogenesis through TGIF/Smad complex-mediated CRMP2 suppression. J Neurosci.

[32] Guilbaud NF, Gas N, Dupont MA, Valette A (1990). Effects of differentiation-inducing agents on maturation of human MCF-7 breast cancer cells. J Cell Physiol.

[33] Kokubun K, Pankajakshan D, Kim MJ, Agrawal DK (2016). Differentiation of porcine mesenchymal stem cells into epithelial cells as a potential therapeutic application to facilitate epithelial regeneration. J Tissue Eng Regen Med.

[34] Kumar P, Luo Y, Tudela C, Alexander JM, Mendelson CR (2013). The c-Myc-regulated microRNA-17~92 (miR-17~92) and miR-106a~363 clusters target hCYP19A1 and hGCM1 to inhibit human trophoblast differentiation. Mol Cell Biol.

[35] Shiras A, Bhosale A, Shepal V, Shukla R, Baburao VS, Prabhakara K (2003). A unique model system for tumor progression in GBM comprising two developed human neuro-epithelial cell lines with differential transforming potential and coexpressing neuronal and glial markers. Neoplasia.

[36] Ying M, Wang S, Sang Y, Sun P, Lal B, Goodwin CR (2011). Regulation of glioblastoma stem cells by retinoic acid: role for Notch pathway inhibition. Oncogene.

[37] Martín-Lorenzo A, Auer F, Chan LN, García-Ramírez I, González-Herrero I, Rodríguez-Hernández G (2018). Loss of Pax5 exploits Sca1-BCR-ABL^p190^ susceptibility to confer the metabolic shift essential for pB-ALL. Cancer Res.

[38] Nguyen PH, Giraud J, Staedel C, Chambonnier L, Dubus P, Chevret E (2016). All-trans retinoic acid targets gastric cancer stem cells and inhibits patient-derived gastric carcinoma tumor growth. Oncogene.

[39] Hasselbach LA, Irtenkauf SM, Lemke NW, Nelson KK, Berezovsky AD, Carlton ET (2014). Optimization of high grade glioma cell culture from surgical specimens for use in clinically relevant animal models and 3D immunochemistry.

[40] Stupp R, Taillibert S, Kanner AA, Kesari S, Steinberg DM, Toms SA (2015). Maintenance therapy with tumor-treating fields plus temozolomide vs temozolomide alone for glioblastoma: a randomized clinical trial. Jama.

[41] Mattoscio D, Chiocca S (2015). SUMO pathway components as possible cancer biomarkers. Future Oncol.

[42] Eifler K, Vertegaal ACO (2015). SUMOylation-mediated regulation of cell cycle progression and cancer. Trends Biochem Sci.

[43] Sharma P, Yamada S, Lualdi M, Dasso M, Kuehn MR (2013). Senp1 is essential for desumoylating Sumo1-modified proteins but dispensable for Sumo2 and Sumo3 deconjugation in the mouse embryo. Cell Rep.

[44] Jiang M, Chiu SY, Hsu W (2011). SUMO-specific protease 2 in Mdm2-mediated regulation of p53. Cell Death Differ.

[45] Meng F, Qian J, Yue H, Li X, Xue K (2016). SUMOylation of Rb enhances its binding with CDK2 and phosphorylation at early G1 phase. Cell Cycle.

[46] Zhou Z, Wang M, Li J, Xiao M, Chin YE, Cheng J (2016). SUMOylation and SENP3 regulate STAT3 activation in head and neck cancer. Oncogene.

[47] Geoffroy MC, Jaffray EG, Walker KJ, Hay RT (2010). Arsenic-induced SUMO-dependent recruitment of RNF4 into PML nuclear bodies. Mol Biol Cell.

[48] Dohmen RJ (2015). SUMO wrestles down myc. Cell Cycle.

[49] Sabò A, Doni M, Amati B (2014). SUMOylation of myc-family proteins. PLoS One.

[50] Wang X, Cunningham M, Zhang X, Tokarz S, Laraway B, Troxell M (2011). Phosphorylation regulates c-Myc's oncogenic activity in the mammary gland. Cancer Res.

[51] Ivanovska I, Ball AS, Diaz RL, Magnus JF, Kibukawa M, Schelter JM (2008). MicroRNAs in the miR-106b family regulate p21/CDKN1A and promote cell cycle progression. Mol Cell Biol.

[52] Wang Z, Liu M, Zhu H, Zhang W, He S, Hu C (2010). Suppression of p21 by c-Myc through members of miR-17 family at the post-transcriptional level. Int J Oncol.

[53] Pandey D, Chen F, Patel A, Wang CY, Dimitropoulou C, Patel VS (2011). SUMO1 negatively regulates reactive oxygen species production from NADPH oxidases. Arterioscler Thromb Vasc Biol.

[54] Medina PP, Nolde M, Slack FJ (2010). OncomiR addiction in an in vivo model of microRNA-21-induced pre-B-cell lymphoma. Nature.

[55] Dimitrova N, Gocheva V, Bhutkar A, Resnick R, Jong RM, Miller KM (2016). Stromal expression of miR-143/145 promotes neoangiogenesis in lung cancer development. Cancer Discov.

[56] Lujambio A, Lowe SW (2012). The microcosmos of cancer. Nature.

[57] Meltzer PS (2005). Cancer genomics: small RNAs with big impacts. Nature.

[58] Cordes KR, Sheehy NT, White MP, Berry EC, Morton SU, Muth AN (2009). miR-145 and miR-143 regulate smooth muscle cell fate and plasticity. Nature.

[59] Mitra AK, Zillhardt M, Hua Y, Tiwari P, Murmann AE, Peter ME (2012). MicroRNAs reprogram normal fibroblasts into cancer-associated fibroblasts in ovarian cancer. Cancer Discov.

[60] Zhang Y, Yang P, Sun T, Li D, Xu X, Rui Y (2013). miR-126 and miR-126* repress recruitment of mesenchymal stem cells and inflammatory monocytes to inhibit breast cancer metastasis. Nat Cell Biol.

[61] Jin X, Jin X, Kim LJY, Dixit D, Jeon HY, Kim EJ (2018). Inhibition of ID1-BMPR2 intrinsic signaling sensitizes glioma stem cells to differentiation therapy. Clin Cancer Res.

[62] Zheng R, Pan L, Gao J, Ye X, Chen L, Zhang X (2015). Prognostic value of miR-106b expression in breast cancer patients. J Surg Res.

[63] Foshay KM, Gallicano GI (2009). miR-17 family miRNAs are expressed during early mammalian development and regulate stem cell differentiation. Dev Biol.

[64] Yang S, Zhang H, Guo L, Zhao Y, Chen F (2014). Reconstructing the coding and non-coding RNA regulatory networks of miRNAs and mRNAs in breast cancer. Gene.

[65] Li BK, Huang PZ, Qiu JL, Liao YD, Hong J, Yuan YF (2014). Upregulation of microRNA-106b is associated with poor prognosis in hepatocellular carcinoma. Diagn Pathol.

[66] Li XP, Zhang XW, Zheng LZ, Guo WJ (2015). Expression of CD44 in pancreatic cancer and its significance. Int J Clin Exp Pathol.

[67] Hermeking H, Rago C, Schuhmacher M, Li Q, Barrett JF, Obaya AJ (2000). Identification of CDK4 as a target of c-MYC. Proc Natl Acad Sci U S A.

[68] Stine ZE, Walton ZE, Altman BJ, Hsieh AL, Dang CV (2015). MYC, Metabolism, and Cancer. Cancer Discov.

[69] O'Donnell KA, Wentzel EA, Zeller KI, Dang CV, Mendell JT (2005). c-Myc-regulated microRNAs modulate E2F1 expression. Nature.

[70] Ogata T, Sano S, Nagata E, Kato F, Fukami M (2012). MAMLD1 and 46, XY disorders of sex development. Semin Reprod Med.

[71] Nicastro G, García-Mayoral MF, Hollingworth D, Kelly G, Martin SR, Briata P (2012). Noncanonical G recognition mediates KSRP regulation of let-7 biogenesis. Nat Struct Mol Biol.

[72] Trabucchi M, Briata P, Garcia-Mayoral M, Haase AD, Filipowicz W, Ramos A (2009). The RNA-binding protein KSRP promotes the biogenesis of a subset of microRNAs. Nature.

[73] Matayoshi S, Chiba S, Lin Y, Arakaki K, Matsumoto H, Nakanishi T (2013). Lysophosphatidic acid receptor 4 signaling potentially modulates malignant behavior in human head and neck squamous cell carcinoma cells. Int J Oncol.

[74] Metzger T, Kleiss C, Sumara I (2013). CUL3 and protein kinases: insights from PLK1/KLHL22 interaction. Cell Cycle.

[75] Powell E, Piwnica-Worms D, Piwnica-Worms H (2014). Contribution of p53 to metastasis. Cancer Discov.

[76] Sinha VC, Qin L, Li Y (2015). A p53/ARF-dependent anticancer barrier activates senescence and blocks tumorigenesis without impacting apoptosis. Mol Cancer Res.

[77] Somasundaram K, Zhang H, Zeng YX, Houvras Y, Peng Y, Zhang H (1997). Arrest of the cell cycle by the tumour-suppressor BRCA1 requires the CDK-inhibitor p21WAF1/CiP1. Nature.

[78] Cheng M, Olivier P, Diehl JA, Fero M, Roussel MF, Roberts JM (1999). The p21(Cip1) and p27(Kip1) CDK 'inhibitors' are essential activators of cyclin D-dependent kinases in murine fibroblasts. Embo j.

[79] Dolan DW, Zupanic A, Nelson G, Hall P, Miwa S, Kirkwood TB (2015). Integrated stochastic model of DNA damage repair by non-homologous end joining and p53/p21-mediated early senescence signalling. PLoS Comput Biol.

[80] Abbas T, Dutta A (2009). p21 in cancer: intricate networks and multiple activities. Nat Rev Cancer.

[81] Vousden KH, Prives C (2009). Blinded by the light: the growing complexity of p53. Cell.

[82] Andorfer P, Rotheneder H (2011). EAPP: gatekeeper at the crossroad of apoptosis and p21-mediated cell-cycle arrest. Oncogene.

[83] Wiese C, Rudolph JH, Jakob B, Fink D, Tobias F, Blattner C (2012). PCNA-dependent accumulation of CDKN1A into nuclear foci after ionizing irradiation. DNA Repair (Amst).

[84] Ren T, Wang Y, Wang C, Zhang M, Huang W, Jiang J (2017). Isolation and identification of human metabolites from a novel anti-tumor candidate drug 5-chlorogenic acid injection by HPLC-HRMS/MS^n^ and HPLC-SPE-NMR. Anal Bioanal Chem.

[85] Erk T, Hauser J, Williamson G, Renouf M, Steiling H, Dionisi F (2014). Structure- and dose-absorption relationships of coffee polyphenols. Biofactors.

[86] Poquet L, Clifford MN, Williamson G (2008). Transport and metabolism of ferulic acid through the colonic epithelium. Drug Metab Dispos.

