# Supplementary material for: Chlorogenic acid effectively treats cancers through induction of cancer cell differentiation
Source: Theranostics. 2019 Sep 19;9(23):6745–63. doi: 10.7150/thno.34674 (PMC6815948; doi:10.7150/thno.34674)
Supplement: Supplementary file 1 — Supplementary figures and tables. [file thnov09p6745s1.pdf]

Supplementary Figures

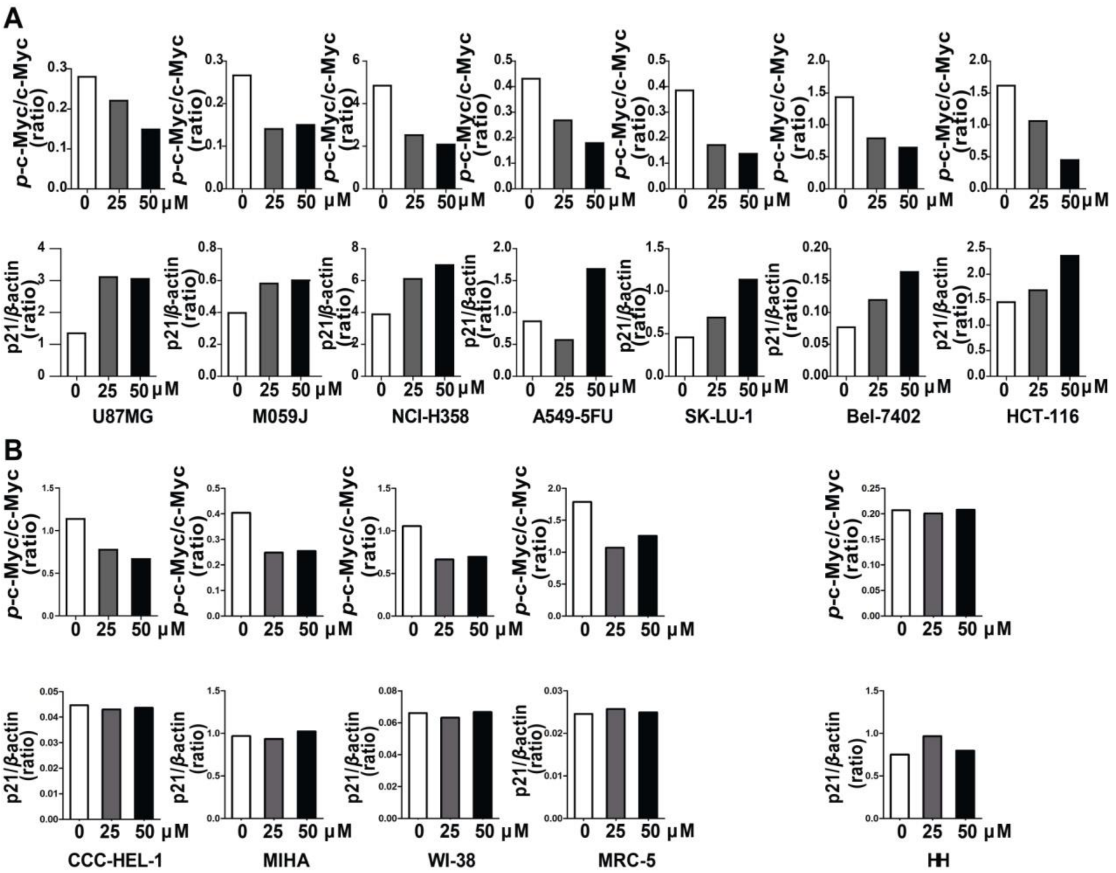

Supplementary Figure S1. CA-caused gene expression changes (density scanning part).

(A) CA-caused gene expression changes in cancer cell lines.

(B) CA-caused gene expression changes in non-cancer cell lines and primary human hepatocytes.

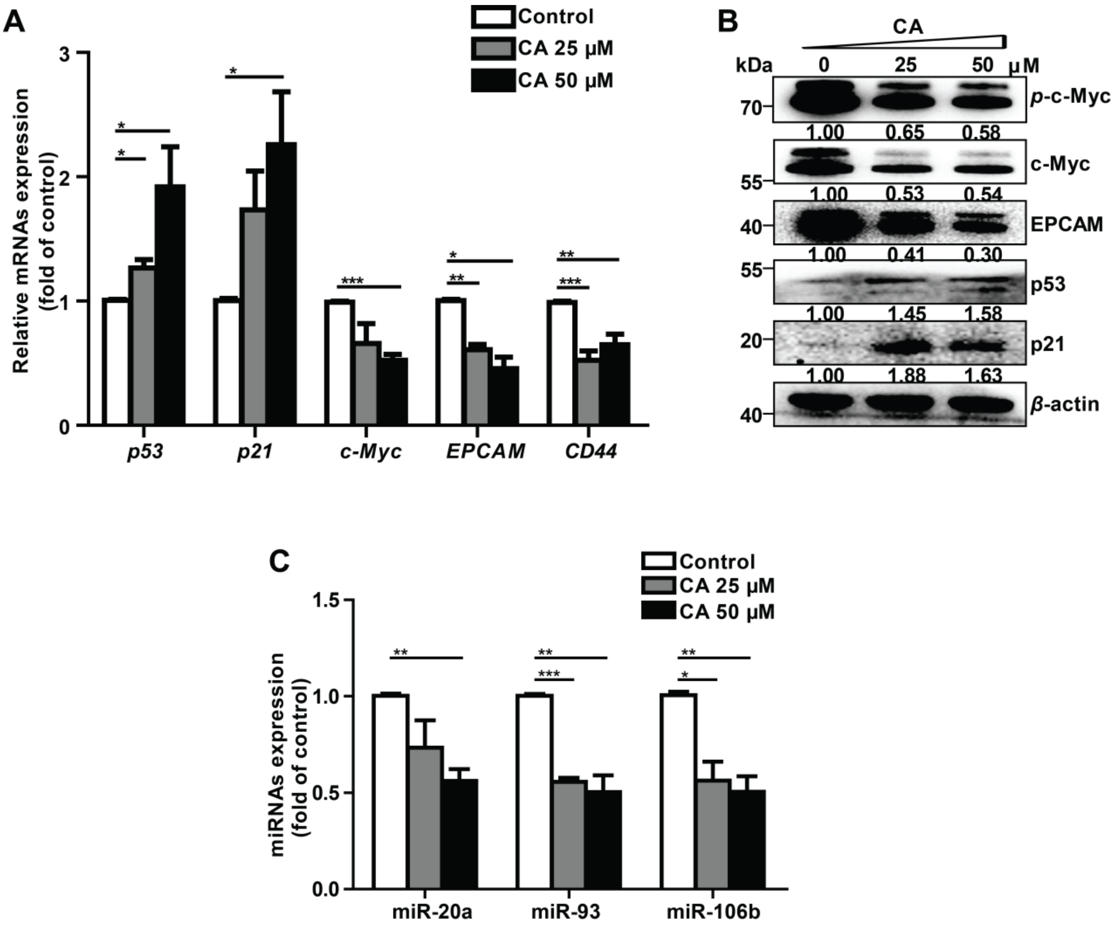

Supplementary Figure S2. CA-induced differentiation of iPS cells.

(A) Human iPS cells were treated with CA (25 or 50 μM), followed by biological tests. mRNA expression of the differentiation-related genes in the iPS cells was assessed by qRT-PCR.

(B) Protein expression in the iPS cells was evaluated by Western blotting and the results were normalized to β-actin in density ratio with the untreated group set as 1.

(C) Expression of miR-17 members in the iPS cells was assessed by qRT-PCR.

All data are presented as mean ± SEM of 3 independent experiments. * $p < 0.05$, ** $p < 0.01$ and *** $p < 0.001$ (CA treated vs untreated control).

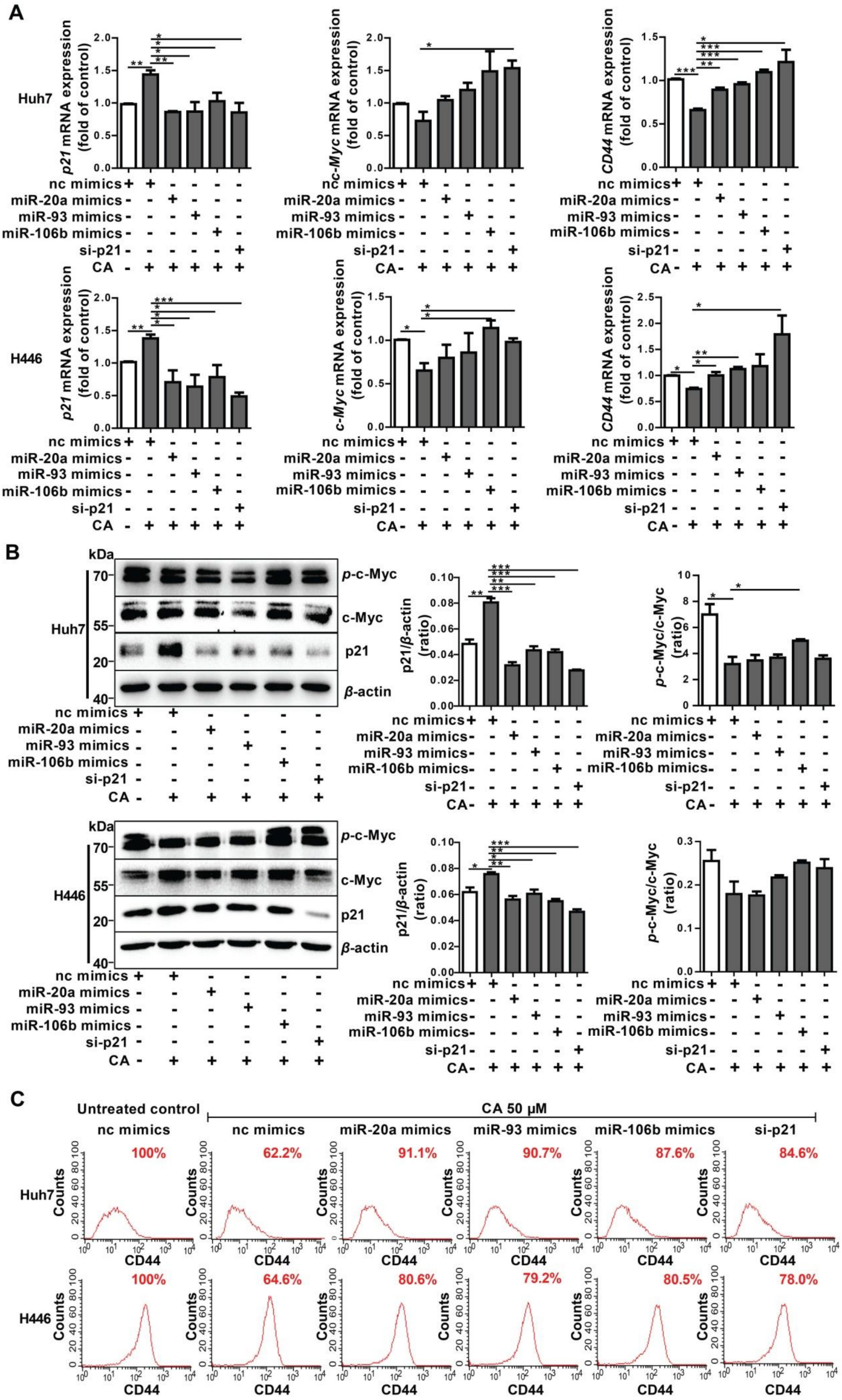
A
Huh7
H446
p21 mRNA expression (fold of control)
c-Myc mRNA expression (fold of control)
CD44 mRNA expression (fold of control)
nc mimics
miR-20a mimics
miR-93 mimics
miR-106b mimics
si-p21
CA
B
kDa
70
55
20
40
p-c-Myc
c-Myc
p21
β-actin
p21/β-actin (ratio)
p-c-Myc/c-Myc (ratio)
C
Untreated control
CA 50 μM
nc mimics
miR-20a mimics
miR-93 mimics
miR-106b mimics
si-p21
100%
62.2%
91.1%
90.7%
87.6%
84.6%
64.6%
80.6%
79.2%
80.5%
78.0%
Counts
CD44

Supplementary Figure S3. Over-expression of miR-17 family members counteracted CA activity through suppressing p21 expression.

Huh7 and H446 cells were treated with CA (50 μM) followed by transfection with negative control (nc, 50 nM), miR-20a/93/106b mimics (50 nM each), or p21 siRNAs (50 nM).

(A) mRNA expression of the differentiation-related genes p21, c-Myc and CD44 in Huh7 (upper) and H446 (lower) cells was examined by qRT-PCR. Transfection of the CA-treated (50 μM) Huh7 or H446 cells with mimics of miR-20a (50 nM), miR-93 (50 nM), miR-106b (50 nM), or p21-siRNAs (si-p21, 50 nM) for 24 h decreased *p21* mRNA levels, but increased *c-Myc* and *CD44* mRNA expression.

(B) Proteins of the differentiation-related genes, p21 and c-Myc, were analyzed in Huh7 (upper) and H446 (lower) cells by Western blotting. The results showed that miR-106b mimics or/and si-p21 partially offset the down-regulatory effect of CA on the level of phosphorylated c-Myc (*p*-c-Myc) protein both in Huh7 and H446 cells. All three mimics and si-p21 significantly abolished the up-regulatory effect of CA on the p21 protein level in Huh7 and H446 cells.

(C) The expression of CD44 in tHuh7 (upper) and H446 (lower) cells was measured by FCM. The mean fluorescence value of the untreated control with negative control mimics (nc mimics) was normalized as 100%. Transfection of miR-20a, -93, -106b mimics and si-p21 abolished the down-regulatory effect of CA on CD44 in Huh7 and H446 cells. Under identical experimental conditions, miR-20a, -93, -106b, or si-p21 elevated the numbers of $CD44^{+}$ cells from 62.2% to 91.1%, 90.7%, 87.6% and 84.6% in Huh7 cells and from 64.6% to 80.6%, 79.2%, 80.5% and 78.0% in H446 cells, respectively.

All data are presented as mean ± SEM of 3 independent experiments. * $p < 0.05$, ** $p < 0.01$ and

*** $p < 0.001$.

Supplementary Table S1. Primers for the qRT-PCR assay.

Genes	Sequence 5'-3'
KHSRP	F; CCACAGCAGGACTACACGAA
	R; GGGGTCTGTCCGTAGTAAGC
p53	F; GGTTGGCTCTGACTGTACCA
	R; TGCGGAGATTCTCTTCCTCT
p21	F; GGCACCTCACCTGCTCTG
	R; GGCGTTTGGAGTGGTAGAAA
c-Myc	F; AAGGCCCCCAAGGTAGTTAT
	R; GCACAAGAGTTCCGTAGCTG
CD44	F; GATCAACAGTGGCAATGGAG
	R; TGCAGGTTCCTTGTCTCATC
EPCAM	F; AAGCACCTGAATTCTCAATGC
	R; GCATCTCACCCATCTCCTTT
GAPDH	F; TCAAGAAGGTGGTGAAGCAG
	R; AGCCAAATTCGTTGTCATACC
